# ﻿Review of the genus *Karschia* Walter, 1889 from Xizang, China (Solifugae, Karschiidae)

**DOI:** 10.3897/zookeys.1204.120164

**Published:** 2024-06-04

**Authors:** Wenlong Fan, Chao Zhang, Feng Zhang

**Affiliations:** 1 Key Laboratory of Zoological Systematics and Application, College of Life Sciences, Hebei University, Baoding, Hebei 071002, China Hebei University Baoding China; 2 Hebei Basic Science Center for Biotic Interaction, Hebei University, Baoding, Hebei 071002, China Hebei University Baoding China

**Keywords:** Camel-spider, COI, taxonomy

## Abstract

The species of the genus *Karschia* Walter, 1889, collected from Xizang, China, were reviewed. A total of six species were recognized using morphological and molecular data, Karschia (Karschia) tibetana Hirst, 1907 is redescribed based on newly collected males and females, and five new species, Karschia (Karschia) dingye**sp. nov.**, Karschia (Karschia) lhasa**sp. nov.**, Karschia (Karschia) zhui**sp. nov.**, Karschia (Karschia) shigatse**sp. nov.**, and Karschia (Karschia) namling**sp. nov.**, are described.

## ﻿Introduction

The family Karschiidae Kraepelin, 1899, comprising four genera and 45 species, is a small family within Solifugae, with a palearctic distribution in north Africa, the Middle East, and central Asia (WSC 2024). Among the four genera within Karschiidae, *Barrus* Simon, 1880, is a monotypic genus; *Barrussus* Roewer, 1928, comprises three species, *Eusimonia* Kraepelin, 1899, comprises 15 species; and *Karschia* Walter, 1899, comprises 26 species (WSC 2024).

The genus *Karschia* is divided into two subgenera: the nominative subgenus, *Karschia* Walter, 1889, found in North Africa, the Middle East, and central Asia, comprising 18 species; and the subgenus Rhinokarschia Birula, 1935, found in central Asia, comprising eight species. Five *Karschia* species have been recorded from China, one *Rhinokarschia* species, Karschia (Rhinokarschia) rhinoceros Birula, 1922 (♂♀, Xinjiang), and four *Karschia* species, Karschia (Karschia) birulae Roewer, 1934 (♂♀, Xinjiang), Karschia (Karschia) tarimina Roewer, 1933 (♀, Xinjiang), Karschia (Karschia) tienschanica Roewer, 1933 (♀, Xinjiang) and Karschia (Karschia) tibetana Hirst, 1907 (♂♀, Xizang) (Harvey 2003). Additionally, Lawrence (1954) described Karschia (Karschia) nubigena, collected by Dr. Noel Humphreys, a medical officer in the British Mount Everest expedition of 1936; the World Solifugae Catalog (WSC) and Harvey’s catalog mention the type locality as Mount Everest, Nepal (Harvey 2003; WSC 2024), but the route followed by that expedition suggests it was more likely collected from the Xizang side (Ruttledge et al. 1936). Based on this, it is believed that Karschia (Karschia) nubigena also occurs in Xizang.

The genus *Karschia* sets itself apart from other genera within Karschiidae easily by possessing a rotatable, long, and rolled flagellum, often described as sessile (Birula 1922, 1938; Bird et al. 2015). This flagellum maintains a core resemblance to a plumose seta, typically adorned with a delicate fringe of setae, which Lawrence (1954) mistakenly referred to as “cilia”. The flagellum of *Karschia* is understood to comprise three components: a distinct stalk, a basal peg sharing similarities with the base of a whip-like flagellum, and an elongated filiform structure resembling a shaft, thus resembling a composite flagellum (Bird et al. 2015). Roewer (1934) described the flagellum of *Karschia* as an elongated double-walled tubular structure, originating from a seta that widened along its length. The margins of the flagellum rolled inward until they nearly formed a closed canal, suggesting a process of longitudinal in-furling. However, it is possible that the hypothesized double membrane was lost. Consequently, the flagellum of *Karschia* resembles a composite flagellum, with identifiable components such as a “stalk”, “base”, and “shaft”, though its sessile nature awaits further investigation (Bird et al. 2015).

The flagellar complex of *Karschia* comprises several structures: a long, typically coiled, filiform flagellum; plumose setae, located ventrally to the flagellum and modified to varying degrees, including broadening referred to as flagellar complex plumose (*fcp*) setae; and one or two acuminate subspiniform setae, usually swollen at the base and situated dorsoproximal to the flagellar attachment point, labeled as flagellar complex subspiniform (*fcs*) setae (Gromov 2004; Bird et al. 2015).

Males of *Karschia* lack spiniform setae on the anterior edge of the propeltidium (on both sides of the ocular tubercle) (Gromov 1998, 2004). Females of this genus exhibit differences, notably in the morphology of the more modified genital segment. The subgenus Karschia differs from the subgenus Rhinokarschia by the absence of a hornlike process on the fixed finger of the chelicerae in males (though a low crest may be present instead), and by the presence of transverse, oval, or triangular-shaped genital sternites in females, which cover the genital opening (Gromov 2004).

The systematics and phylogenetic relationships of *Karschia* remain poorly understood, as its congeners are relatively local and rare species (Gromov 2004). Species diagnoses in this genus are mainly based on male characters. Female descriptions are limited to body size and coloration, chelicerae dentition, and the shape and number of ctenidia on the fourth abdominal segment. The female genital segment has previously been considered of little or no taxonomic significance in species diagnosis (Roewer 1933; Birula 1938). This makes it difficult to identify female specimens. In order to address the issues related to female identification, Gromov (2004) proposed that for female diagnosis, the shape and size of the ctenidia on the fourth abdominal segment, as well as the shape, size, and relative arrangement of the sclerites of the genital segment are more reliable than others, i.e., the chelicerae dentition, the number of ctenidia on the fourth abdominal segment, the body size and coloration.

Xizang Autonomous Region, located in the southwestern part of the Qinghai-Xizang Plateau and known as the “Roof of the World,” has an average elevation exceeding 4,000 meters. The high altitude and unique geographical location form its distinctive climate and rich biodiversity, including many rare and endangered species. Xizang has become a hotspot and crucial area for global biodiversity research in recent years. However, research on Solifugae in Xizang has been limited, with only four species previously reported: *Galeodescaspius* Birula, 1890, *Karschiatibetana* Hirst, 1907, *Karschianubigena*Lawrence 1954, and *Triditarsustibetanus* Roewer, 1933.

During our biodiversity survey, we revealed a widespread distribution of Solifugae in Xizang. During the process of identifying collected specimens, we observed a high level of diversity within the genus *Karschia* in Xizang through comparison with diagnoses, descriptions, and drawings in original literature of all known species. This research used morphological characters and molecular data to investigate the taxonomy of Solifugae in Xizang. To solve the problem of male and female combinations and to ensure the precision of our morphological identifications, we carried out genetic distance comparisons on suspected new species. For each species, both a male and a female specimen were chosen to extract genomic DNA and the COI gene was amplified. In conclusion, comparisons of morphological characteristics and molecular genetic distances have led us to conclude that there are seven species of *Karschia* distributed in Xizang.

From the study of the newly collected material, we provide a redescription of Karschia (Karschia) tibetana Hirst, 1907 based on males and females collected from the type locality, and the description of five new species: Karschia (Karschia) dingye sp. nov., Karschia (Karschia) lhasa sp. nov., Karschia (Karschia) zhui sp. nov., Karschia (Karschia) shigatse sp. nov., and Karschia (Karschia) namling sp. nov.

## ﻿Materials and methods

The specimens were collected during the day by hand from under stones, and preserved in 75% and 95% alcohol, respectively. Photographs were taken using a Leica M205A stereomicroscope equipped with a DFC 550 CCD and an Olympus BX51 microscope equipped with a Kuy Nice CCD camera and were imported into Helicon Focus v. 7 for stacking. Plates and photographs were edited and retouched using Adobe Photoshop 2022. Drawings was made using the Inkscape software (v. 1.0.2.0). All measurements are in mm. Pedipalp measurements are shown as: total length (femur, tibia, metatarsus, tarsus); leg measurements are shown as: total length (femur, tibia, metatarsus, tarsus, claw). All specimens are deposited in the Museum of Hebei University (**MHBU**), Baoding, China.

Descriptions follow the format of Birula (1938), with some modifications by Gromov (2004). The terminology used for identifying teeth and other structures in the chelicerae follows Bird et al. (2015). Identification of individual teeth used Bird et al.’s criteria (2015) for primary homology assessment of dentition. The term ‘ctenidia’ stands for long, single-tipped (non-bifid) and flexible setiform structures present on some opisthosomal sternites.

The QIAGEN DNeasy Blood & Tissue Kit (Qiagen Inc., Valencia, CA) was used to extract genomic DNA from the muscle tissues of the legs for one male and one female of each species. The PCR primer for a partial fragment of the mitochondrial cytochrome oxidase subunit (COI) gene is the universal primer for invertebrate DNA barcoding LCO1490 and HCO2198 (Folmer et al. 1994). All sequences were analyzed using BLAST and are deposited in GenBank (Table 1). Sequence alignment was performed in MAFFT v. 7.313. The p-distance of intra-specific nucleotide divergence was calculated in MEGA.11.0.

**Table 1.** Voucher specimen information.

	Species	Sex	GenBank accession number	Sequence length
**1**	*K.tibetana*	Male	PP587316	685
**2**	*K.tibetana*	female	PP594087	687
**3**	*K.zhui* sp. nov.	Male	PP600574	687
**4**	*K.zhui* sp. nov.	Female	PP600573	696
**5**	*K.shigatse* sp. nov.	Male	PP600575	687
***6***	*K.shigatse* sp. nov.	Female	PP600578	683
**7**	*K.dingye* sp. nov.	Male	PP600577	687
**8**	*K.dingye* sp. nov.	Female	PP600576	670
**9**	*K.namling* sp. nov.	Male	PP600579	687
**10**	*K.namling* sp. nov.	Female	PP600580	688
**11**	*K.lhasa* sp. nov.	Male	PP600581	683
**12**	*K.lhasa* sp. nov.	Female	PP600582	682

Abbreviations as follows: A/CP is the sum of the lengths of pedipalp, leg I, and leg IV divided by the sum of the lengths of chelicera and propeltidium, indicating the length of appendages in relation to body size. Long-legged species have larger A/CP ratios. **CL/CH**, chelicera length/height, large CL/CH ratios suggest a narrow cheliceral morphology, while a more robust morphology is represented by a smaller ratio. **CL**, chelicera length; **CH**, chelicera height; ***fcp*** (modified *pvd*) , flagellar complex plumose setae; **fcs**, flagellar complex subspiniform to spiniform setae; **FD**, fixed finger, distal tooth; **FM**, fixed finger, medial tooth; **FP**, fixed finger, proximal tooth; **FSD**, fixed finger, subdistal tooth/teeth; **FSM**, fixed finger, submedial tooth/teeth; ***pdp***, prodorsal proximal setae; **PF**, profondal teeth; **PFM**, profondal medial tooth/teeth; **PFP**, profondal proximal tooth/teeth; **PFSP**, profondal subproximal tooth/teeth; **PH**, Propeltidium height; **PL**, Propeltidium length; ***pvd***, proventral distal setae; ***pvsd***, proventral subdistal setae; **MM**, movable finger, medial tooth; **MP**, movable finger, proximal tooth; **MSM**, movable finger, submedial tooth/teeth; **MSP**, movable finger, subproximal tooth/teeth; **MST**, movable finger, subterminal tooth/teeth; **RF**, retrofondal teeth; **RFA**, retrofondal apical tooth/teeth; **RFM**, retrofondal medial tooth/teeth; **RFP**, retrofondal proximal tooth/teeth; **RFSM**, retrofondal submedial tooth/teeth; **RFSP**, retrofondal subproximal tooth/teeth; ***rlf***, retrolateral finger setae; ***sme***, socket margin elevation.

## ﻿Results of genetic analyses

In this study, genomic DNA was extracted from one male and one female of each species, and genetic distances were analyzed. The intraspecific genetic distance ranged from 0% to 2.20%, while the interspecific genetic distance varied from 8.08% (between *K.shigatse* sp. nov. (female) and *K.dingye* sp. nov. (male)) to 12.92% (*K.shigatse* sp. nov. (male) and *K.lhasa* sp. nov. (male)) (Table 2). The average genetic distance ranged from 0% to 7.92% within *Galeodescaspius* Birula, 1890 (Maddahi et. al 2016). Therefore, based on significant morphological differences and genetic distance, we conclude that the six species in this study can be distinguished effectively.

**Table 2.** Genetic distance among the six species.

	*K.tibetana*	*K.tibetana*	*K.zhui*	*K.zhui*	*K.shigatse*	*K.shigatse*	*K.dingye*	*K.dingye*	*K.namling*	*K.namling*	*K.lhasa*	*K.lhasa*
*K.tibetana*												
*K.tibetana*	0.88%											
*K.zhui*	11.45%	11.16%										
*K.zhui*	11.31%	11.01%	0.15%									
*K.shigatse*	10.57%	10.28%	10.87%	10.72%								
*K.shigatse*	11.16%	10.57%	10.57%	10.57%	2.20%							
*K.dingye*	12.79%	12.50%	9.71%	9.85%	8.53%	8.09%						
*K.dingye*	12.99%	12.69%	9.82%	9.97%	8.61%	8.16%	0.30%					
*K.namling*	8.52%	8.22%	11.31%	11.45%	11.60%	11.60%	11.91%	12.39%				
*K.namling*	8.52%	8.22%	11.31%	11.45%	11.60%	11.60%	11.91%	12.39%	0%			
*K.lhasa*	12.33%	12.04%	11.60%	11.75%	12.92%	12.33%	10.59%	10.88%	12.78%	12.78%		
*K.lhasa*	12.33%	12.04%	11.60%	11.75%	12.92%	12.33%	10.59%	10.88%	12.78%	12.78%	0%	

### ﻿Taxonomy

**Family Karschiidae Kraepelin, 1899**

**Genus *Karschia* Walter, 1889**

#### ﻿ Karschia (Karschia) tibetana

Taxon classificationAnimaliaSolifugaeKarschiidae

Hirst, 1907

2C7C03B1-99E7-5FB8-9369-0C6BA25E39CF

Figs 1 , 3A–D , 6A, B , 8A–D , 11A , 12A , 13A–D , 16A, B , 17A , 18A , 19A, C , Tables 1 , 2

Karschia tibetana : Hirst, 1907a: 322–324, figs 1, 2; Hirst, 1912: 233–234; Birula, 1922: 197; Roewer, 1932: n/a, figs 110c, 143a, 143a; Roewer, 1933: 298, figs 221a, 222a, 223o; Zilch, 1946: 123.
Karschia (Karschia) tibetana Hirst: Harvey, 2003: 286.

##### Type material.

***Holotype*** ♂, China: Xizang, Shigatse Prefecture, Gyangze County, stored at **NHMUK** (of Natural History Museum, United Kingdom), not examined. ***Paratypes***: 6♂♂, 9♀♀, China: Xizang, Gyangze, Kamba-Dzong, Tinki, Shekar, Kyishong, stored at SMF (Naturmuseum und Forschungsinstitut Senckenberg, Frankfurt am Main), not examined.

**Figure 1.**
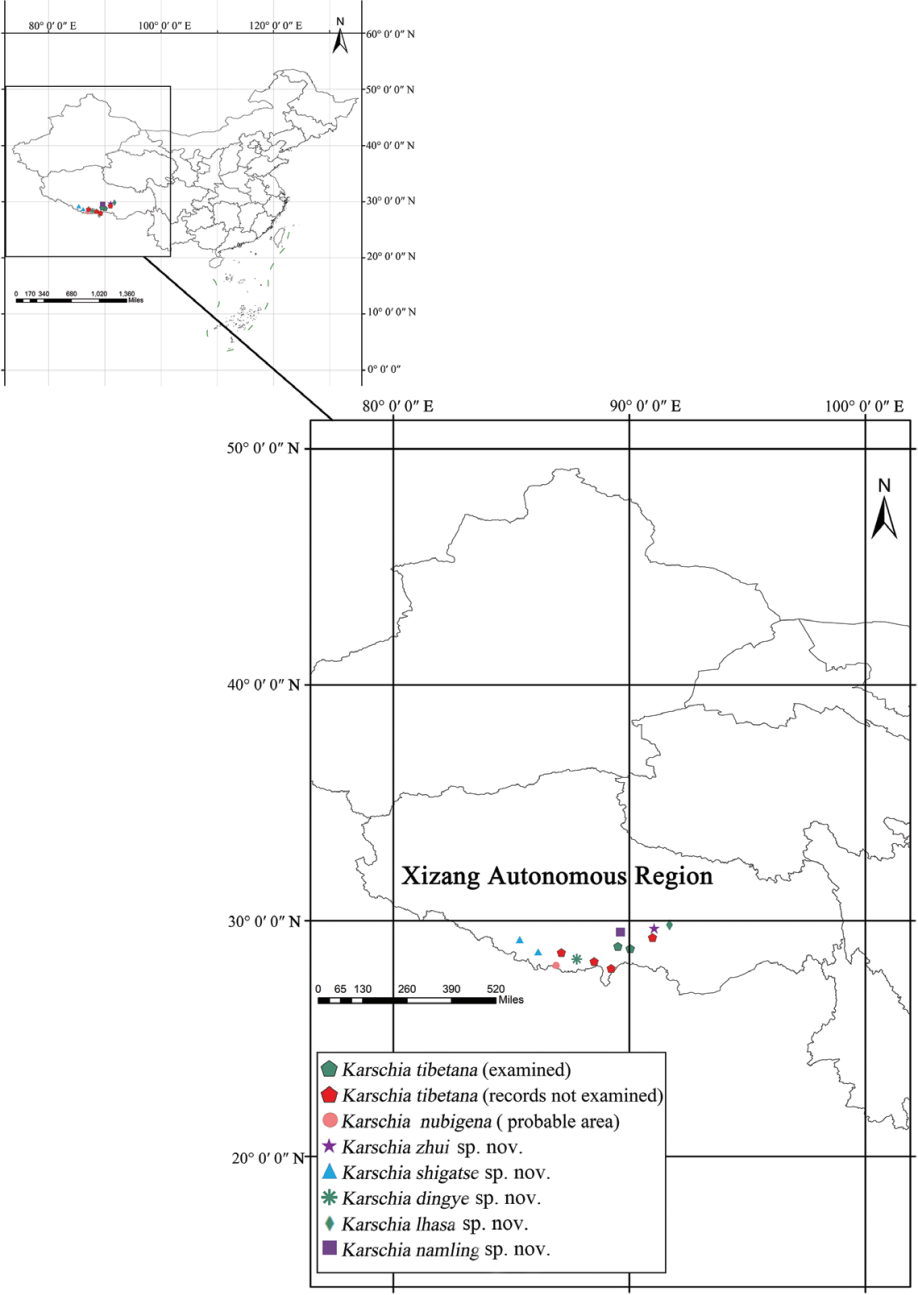
Map plotting known locality records.

**Figure 2.**
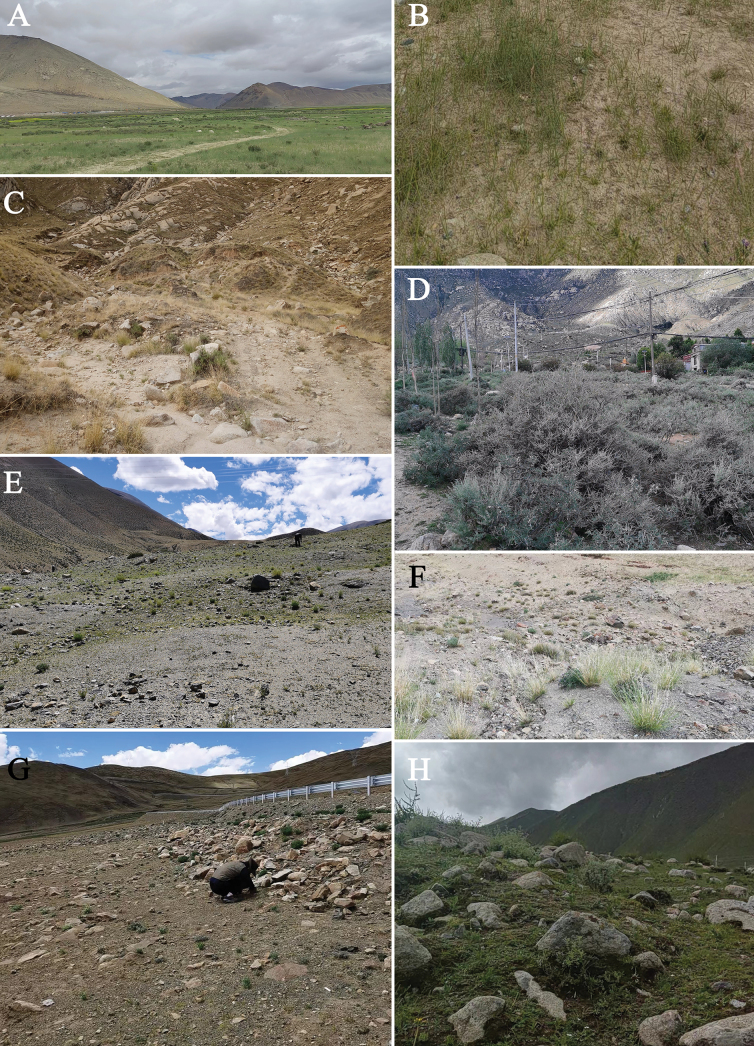
Habitat where *K.dingye* sp. nov. (**A, B**), *K.lhasa* sp. nov. (**C**), *K.shigatse* sp. nov. (**E–G**) and *K.namling* sp. nov. (**H**) have been found. **A, B** Xizang, Dingye County, Gyangkar Town **C** Xizang, Lhasa City, Maizhokunggar County **D** Xizang, Lhasa City, Drepung Monastery **E, F** Xizang, Nyalam County, Mainqu Town **G** Xizang, Gyirong County, Zheba Town **H** Xizang, Namling County, Nubma Town.

**Figure 3.**
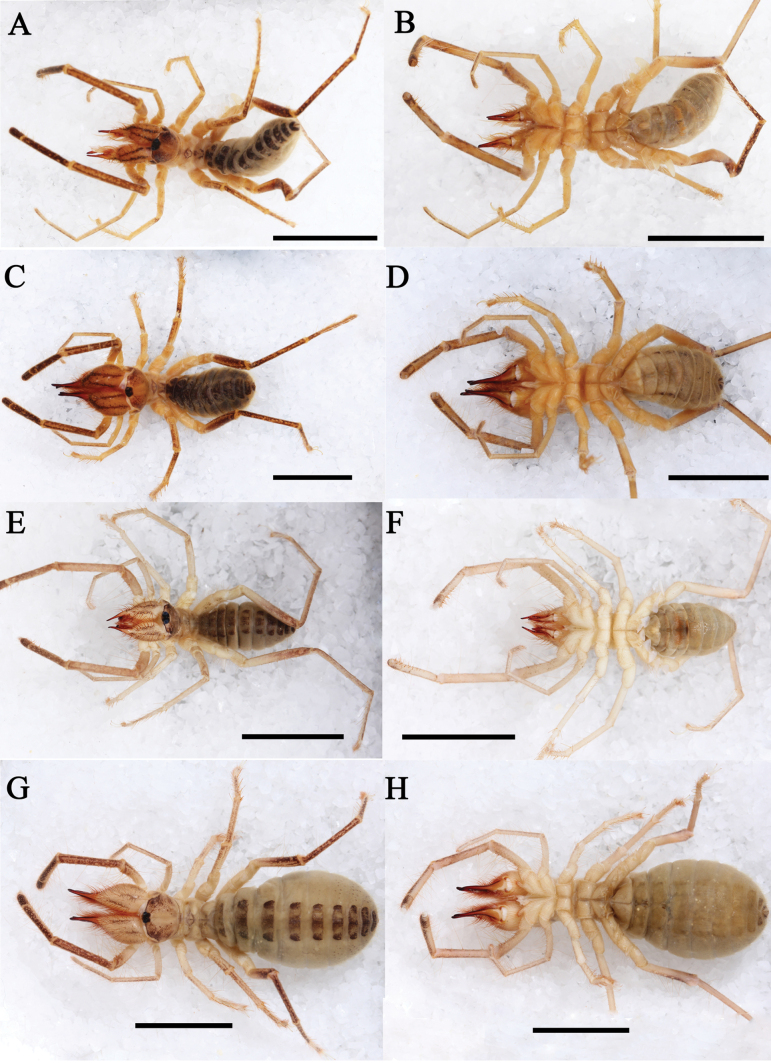
Habitus **A–D***K.tibetana*, habitus, male (**A, B**) and female (**C, D**) **E–H***K.dingye* sp. nov. habitus, male (**E, F**) and female (**G, H**). Scale bars: 8 mm.

**Figure 4.**
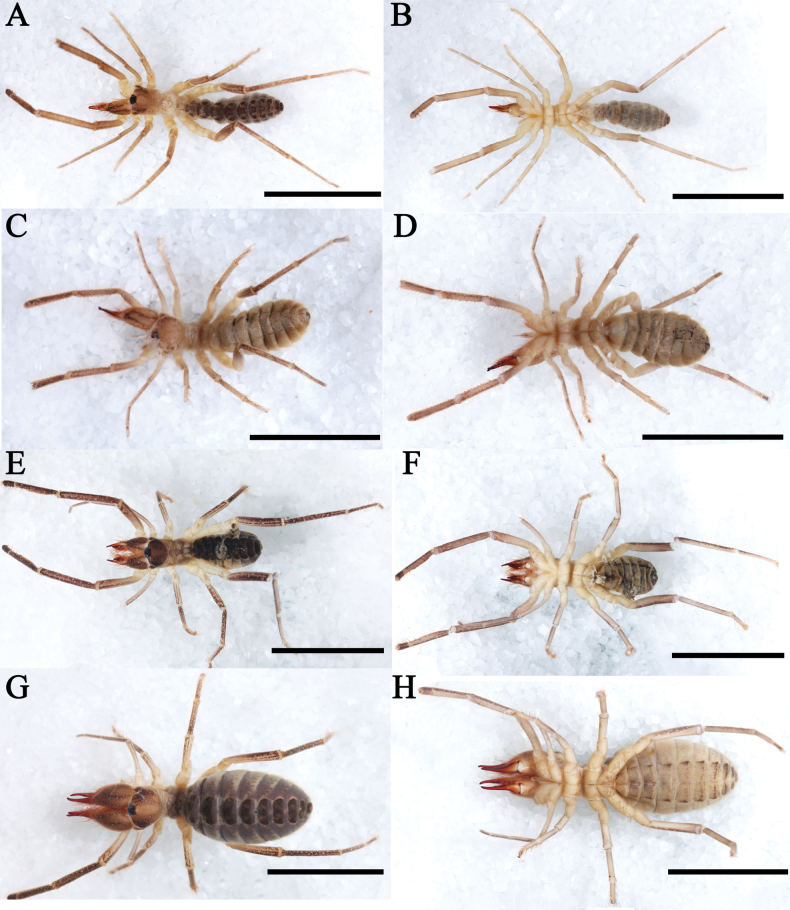
Habitus **A–D***K.lhasa* sp. nov., habitus, male (**A, B**) and female (**C, D**) **E–H***K.zhui* sp. nov., habitus, male (**E, F**) and female (**G, H**). Scale bars: 10 mm.

**Figure 5.**
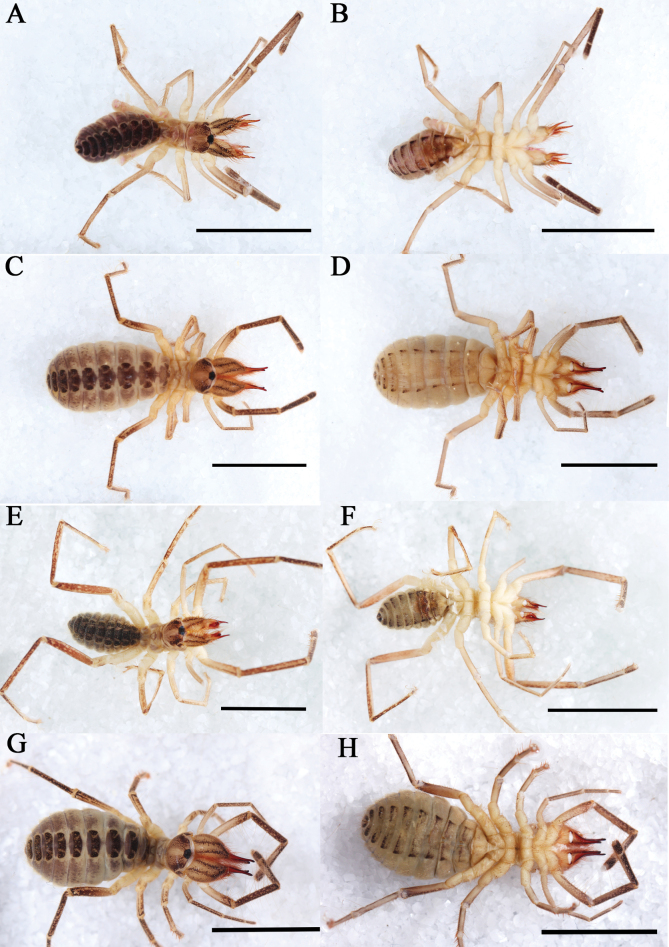
Habitus **A–D***K.shigatse* sp. nov., habitus, male (**A, B**) and female (**C, D**) **E–H***K.namling* sp. nov., habitus, male (**E, F**) and female (**G, H**). Scale bars: 10 mm.

##### Materials examined.

4♂♂ (MHBU-Sol-XZ2014080601–04), 3♀♀ (MHBU-Sol-XZ2014080605–08), China: Xizang, Shigatse Prefecture, Gyangze County, Enzha Village, 6.VIII.2014, leg. Chao Zhang; 1♂ (MHBU-Sol-XZ2018070901), China: Xizang, Shigatse Prefecture, Gyangze County, Ralung Town, 28.8176°N, 90.0369°E, 4451 m elev., 9.VII.2018, leg. Yannan Mu.

##### Diagnosis.

*K.tibetana* differs from all *Karschia* species except *K.nubigena*, *K.dingye* sp. nov., *K.lhasa* sp. nov., *K.zhui* sp. nov., *K.shigatse* sp. nov. and *K.namling* sp. nov. by male cheliceral movable finger with MSM teeth. *K.tibetana* differs from *K.nubigena* by having fringed flagellum (Fig. 11A) and male ctenidia in sternite IV cylindrical and not wide at bottom (Fig. 19C), from *K.dingye* sp. nov., *K.lhasa* sp. nov. and *K.zhui* sp. nov. by pedipalpal metatarsus without or with only a few papillae (Fig. 16B), and from *K.shigatse* sp. nov. and *K.namling* sp. nov. by flagellum without lateral apophysis and long *fcp* (Fig. 11A). The female genital operculum is easily recognizable in all known species; it is long, subtriangular, and with no clear demarcation between the plates, with the rear edge being lightly chitinized (Fig. 17A).

##### Redescription.

**Male** (MHBU-Sol-XZ2014080601).

***Measurements*.** Total body length 17.86, CL 4.61, CH 1.64, PL 2.14, PW 2.72, A/CP 8.28, CL/CH 2.81. Pedipalp 18.77 (5.34, 6.13, 3.81, 0.96), Leg I 14.70 (3.33, 3.68, 2.70, 1.30, 0.09), Leg II 11.49 (2.12, 2.81, 2.00, 0.85, 0.93), Leg III 15.07 (3.51, 3.91, 2.18, 0.55, 0.88), Leg IV 22.366 (5.07, 5.76, 3.60, 1.30, 1.28).

***Coloration*.** In 75% ethanol-preserved specimens (Fig. 3A, B). The general background deep yellow. Opisthosoma gray-yellow, with black tergites and pale black sternites. Propeltidium tinged pale brown. Ocular tubercle black. Mesopeltidium and metapeltidium with special black stripes. Chelicerae with manus predominantly yellowish with some black areas, and a retrolateral view of chelicerae with three black longitudinal stripes. Pedipalps and legs yellow, legs III and legs IV tinged with pale brown on distal regions of femora and proximal parts of tibiae. Proximal regions of the pedipalpal femur, tibia, metatarsus, and tarsus tinged with brown. Malleoli yellow.

**Figure 6.**
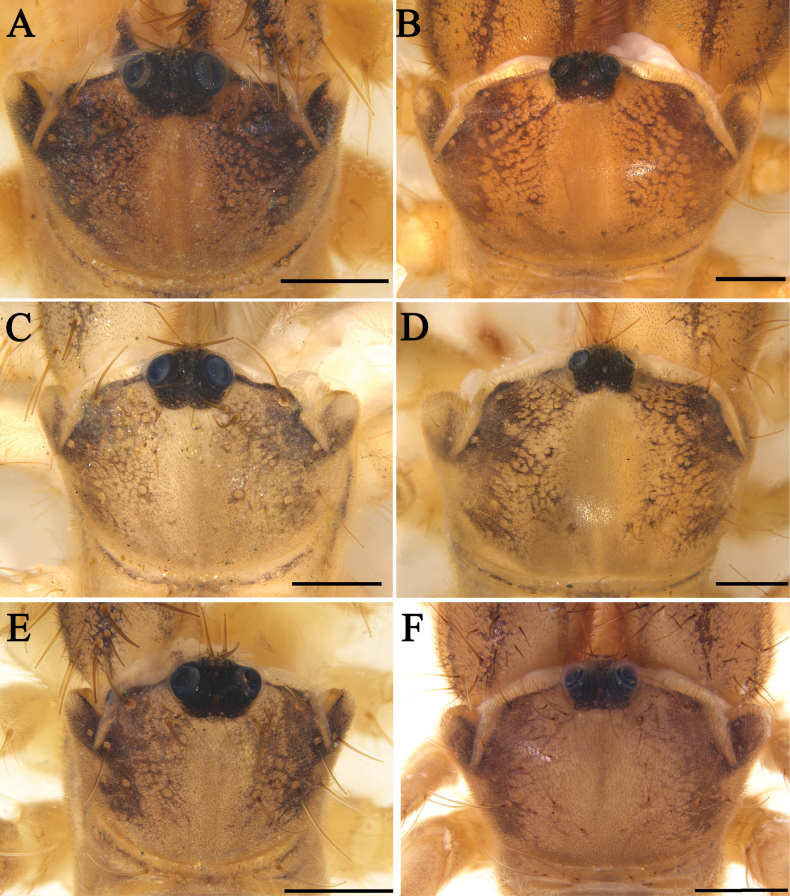
Propeltidium **A***K.tibetana*, male **B***K.tibetana*, female **C***K.dingye* sp. nov., holotype male **D***K.dingye* sp. nov., paratype female **E***K.lhasa* sp. nov., holotype male **F***K.lhasa* sp. nov., paratype female. Scale bars: 1 mm.

***Propeltidium*.** Wider than long, with dense pubescence of thin, short, anteriorly directed setae. Anterior, posterior, and lateral edges with several long, curved spiniform setae perpendicular to the surface of the propeltidium. Ocular tubercle with two short and two long middle distal spiniform setae, one long middle spiniform setae, two short spiniform setae, and numerous shorter, thinner, proximal setae (Fig. 6A).

**Figure 7.**
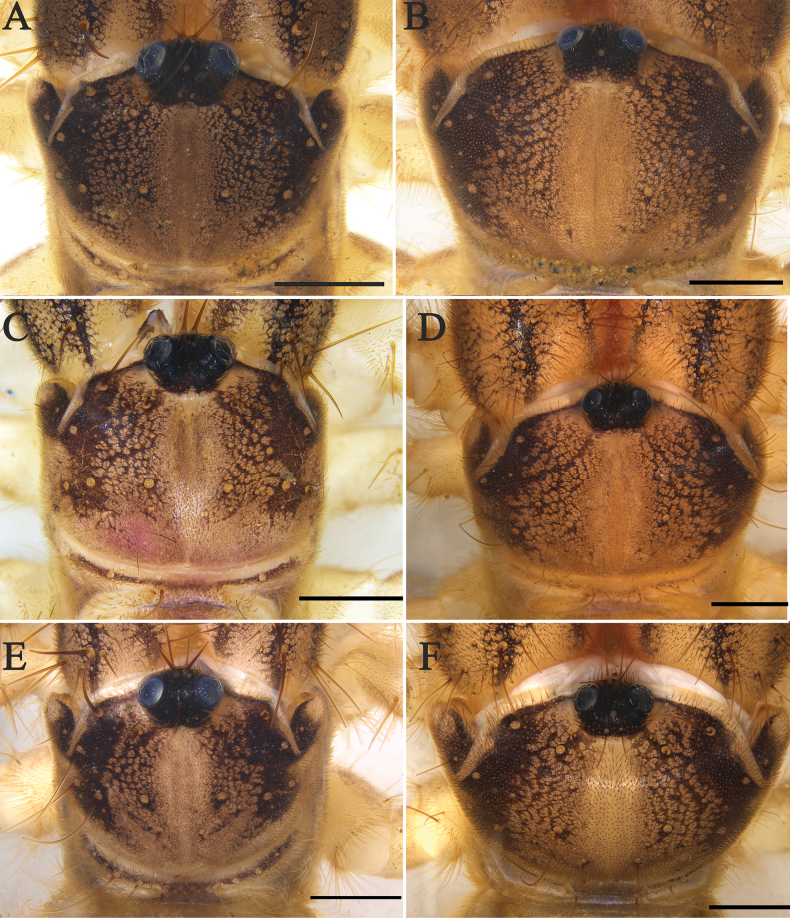
Propeltidium **A.***K.zhui* sp. nov., holotype male **B.***K.zhui* sp. nov., paratype female **C.***K.shigatse* sp. nov., holotype male **D.***K.shigatse* sp. nov., paratype female **E.***K.namling* sp. nov., holotype male **F***K.namling* sp. nov., paratype female. Scale bars: 1 mm.

**Figure 8.**
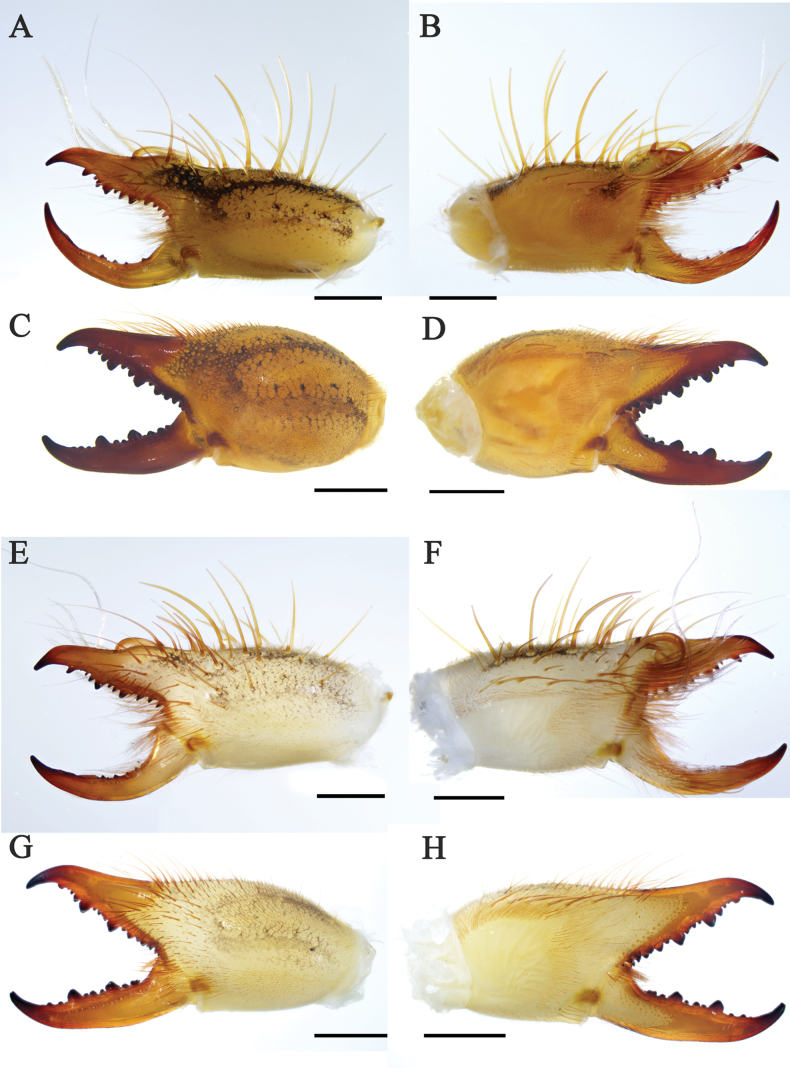
Retrolateral (left) and prolateral (right) cheliceral views **A, B***K.tibetana*, male **C, D***K.tibetana*, female **E, F***K.dingye* sp. nov., holotype male **G, H***K.dingye* sp. nov., paratype female. Scale bars: 1.0 mm (**A, B; E–H**); 2.0 mm (**C, D**).

***Chelicerae*.** Fixed finger primary teeth graded as FP ≈ FD < FM. Profondal teeth series with three tiny teeth; retrofondal teeth series with seven teeth. Dental formulation of fixed finger: FD-(2)-FM-(2)-FP-(7RF) (3PF). Movable finger MP tooth about the same size as MM. Movable finger dental formula: MM-(2)-MP, with two MSM teeth and two MSP (Figs 8A, 13A). Flagellum coiled, fringed and sessile, without lateral apophysis. The flagellar complex includes two long *fcp* and two short, thick *fcs* (Figs 8B, 11A, 13B). Retrolateral and dorsal surfaces of the manus with large, bifurcated tip setae and short simple tip bristle-like setae; retrolateral and dorsal surfaces of the fixed finger with simple tip setae of different sizes. Retrolateral setose area reaching the FSM teeth; prolateral surface with an array of setal types (Figs 8A, B, 13A, B).

**Figure 9.**
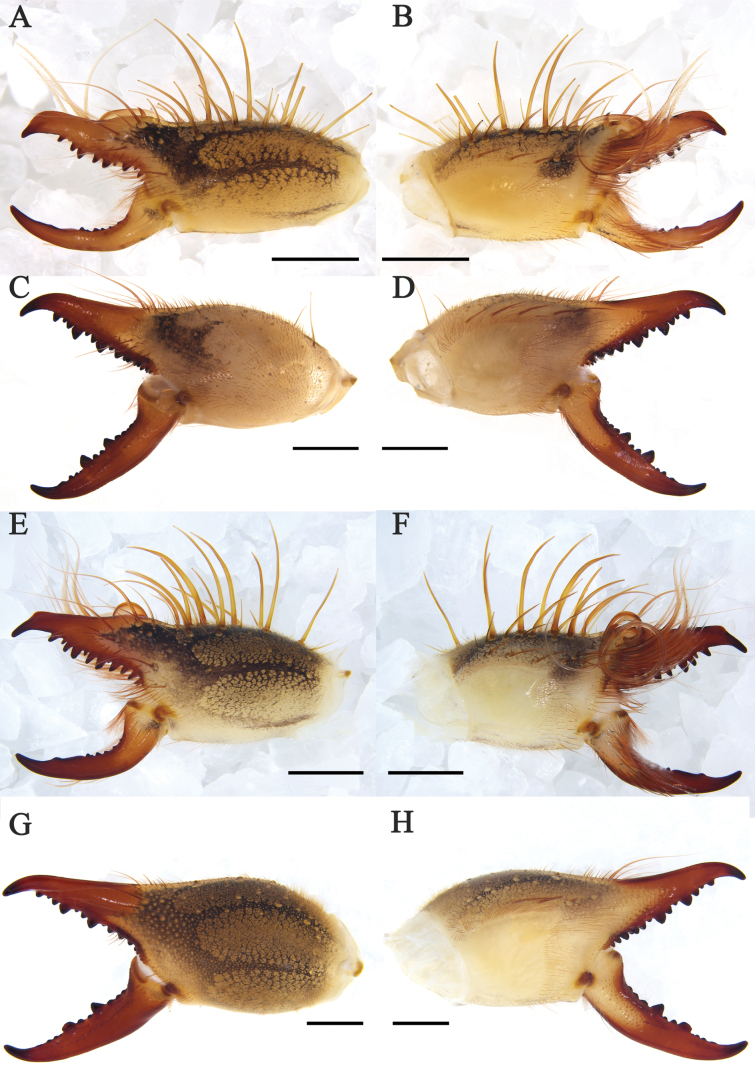
Retrolateral (left) and prolateral (right) cheliceral views **A, B***K.lhasa* sp. nov., holotype male **C, D***K.lhasa* sp. nov., paratype female **E, F***K.zhui* sp. nov., holotype male **G, H***K.zhui* sp. nov., paratype female. Scale bars: 1.0 mm.

**Figure 10.**
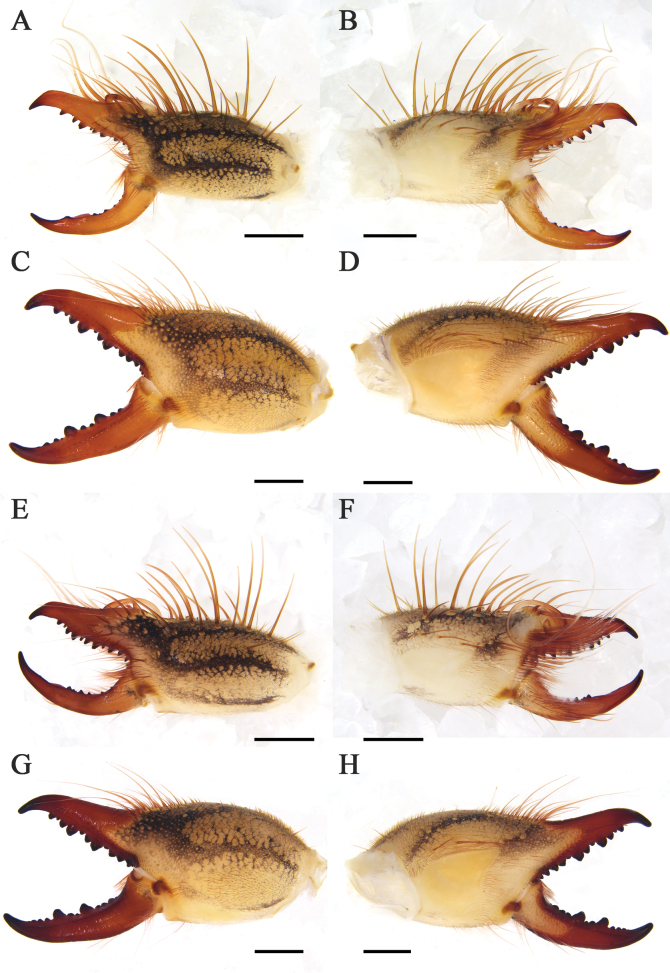
Retrolateral (left) and prolateral (right) cheliceral views **A, B***K.shigatse* sp. nov., holotype male **C, D***K.shigatse* sp. nov., paratype female **E, F***K.namling* sp. nov., holotype male **G, H***K.namling* sp. nov., paratype female. Scale bars: 1.0 mm.

***Opisthosoma*.** The entire surface covered with almost adpressed setae, and numerous long, curved, bifurcate setae. Sternite III with two posterior paramedian groups of ctenidia, being gradually larger to posterior (Fig. 19A); sternite IV with a row of 19 long and thin cylindrical ctenidia (Fig. 19C).

***Pedipalps*.** Entirely covered with short setae and long, thick setae. Tarsus with ten ventral spines; metatarsus with six ventral spines not arranged in pairs and without papillae (Figs 16A, B).

***Legs*.** Entirely covered with long, thick, setae and short setae. Leg I with no spines and two small claws. Tibiae II, III, and IV with a pair of distal spines ventrally, and tibiae II and III with a single dorsal spine. Metatarsus II and III with a series of three dorsal spines, a pair of distal spines ventrally, and some paired short, thick, spine-shaped bristles over their entire ventral surface; metatarsus IV also with these paired bristles over its entire ventral surface and two distal spines ventrally.

**Female** (MHBU-Sol-XZ2014080605).

***Measurements***. Total body length 20.14, CL 7.35, CH 2.93, PL 4.15, PW 2.91, A/CP 4.66, CL/CH 2.50. Pedipalp 17.34 (3.82, 5.25, 4.04, 1.15), Leg I 14.62 (3.04, 4.00, 2.17, 1.22, 0.15), Leg II 11.93 (2.00, 2.70, 1.72, 0.84, 0.98), Leg III 14.97 (2.66, 3.36, 2.40, 0.75, 1.04), Leg IV 21.55 (4.16, 6.06, 3.44, 1.49, 1.10).

***Coloration*.** In 75% ethanol-preserved specimens (Fig. 3C, D). Coloration as in the males.

***Propeltidium*.** Much longer than wide with a dense pubescence of thinner, short, anteriorly directed setae. Anterior, posterior, and lateral edges with several long, curved spiniform setae that are perpendicular to the surface of the propeltidium. Ocular tubercle with some long setae and numerous shorter, thinner setae (Fig. 6B).

***Chelicerae*.** Dental formulation of fixed finger: FD-(2)-FM-(2)-FP-(7RF) (5PF). Dental formulation of movable finger: MM-(2)-MP, with four MST and four MSP. Fondal teeth graded as II, III, IV, I, tiny V, tiny VI, tiny VII retrolaterally; II, I, III, tiny IV, tiny V prolaterally (Figs 8C, D, 12A, 13C, D).

***Opisthosoma*.** The entire surface covered with almost adpressed setae and numerous long, curved, bifurcate setae. Genital operculum long subtriangular and bottom widened (with lightly chitinized folds) between and behind them (Fig. 17A). Sternite IV with eight ctenidia on each side, for a total of 16 longer acicular ctenidia extending 1/2 length of the succeeding sternite (Fig. 18A).

***Pedipalps*.** Entirely covered with short setae and long, thick setae, without spines.

***Legs*.** As in the males.

##### Variability.

Males. Total length 16.17–20.35. Body coloration pale yellow to tan. Chelicerae with manus yellowish to brown. Pedipalps without or with only a few papillae. The number of cheliceral fixed finger fondal teeth 9–11 (profondal teeth 3–4; retrofondal teeth 6–7). The number of ctenidia on sternite IV 18–20. Pedipalp tarsus with 9–11 spines, metatarsus with 5–7 spines. Females. Total length 19.53–22.36. Variability of body coloration as in males. The number of cheliceral fixed finger fondal teeth 10–13 (profondal teeth 4–5; retrofondal teeth 6–8). MST 3–4, MSP 4–5. The number of ctenidia on sternite IV 16–18.

##### Distribution and Habitat.

China (Xizang). Habitat: high altitude meadow and semi-desert meadow.

##### Remark.

In the original description, *K.tibetana* flagellum was described as smooth with small lateral apophysis (Hirst 1907). However, upon re-examination by Hirst (1912), it was found that the flagellum did not have a small lateral apophysis. Roewer (1933) reexamined the holotype and confirmed that the flagellum is fringed, not smooth. Based on my examination of specimens collected from the type locality, the flagellum of *K.tibetana* is fringed, and without small lateral apophysis. Based on the comparison of genetic distances, with a genetic distance of 0.88% (Table 2) between male and female collected from same locations, we believe that they are same species.

#### ﻿ Karschia (Karschia) dingye sp. nov.

Taxon classificationAnimaliaSolifugaeKarschiidae

E1D0709B-C67B-5D9F-9814-61CE9D9B872D

https://zoobank.org/CFF26FE4-7DF2-48B8-B670-5C202CE715D2

Figs 1 , 3E –H , 6C, D , 8E –H , 11B , 12B , 13E –H , 16C, D , 17B , 18B , 19B, D , Tables 1 , 2

##### Type material.

***Holotype*** ♂ (MHBU-Sol-XZ2023072701), China: Xizang, Shigatse Prefecture, Dingye County, Gyangkar Town, 28.3702°N, 87.7732°E, ca 4200 m elev., 27.VII.2023, leg. Yanmeng Hou, Zhiyong Yang. ***Paratypes***: 25♂♂ (MHBU-Sol-XZ2023072702–27), 15♀♀ (MHBU-Sol-XZ2023072728–43), with same data as holotype.

##### Etymology.

Noun in apposition taken from Dingye County where this species was collected.

##### Diagnosis.

**Figure 11.**
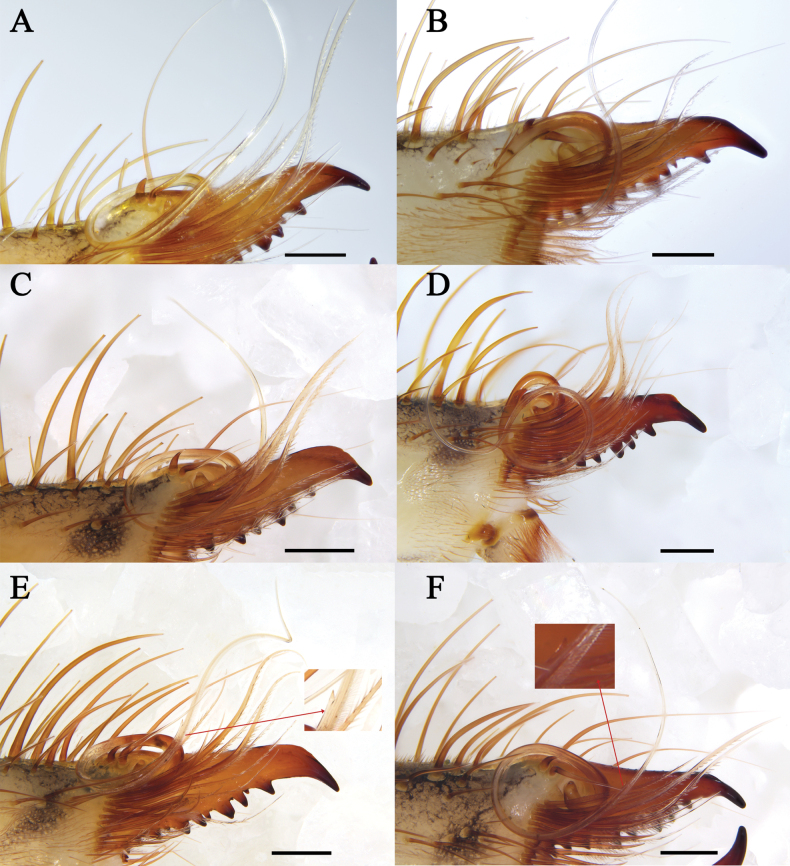
Flagellum **A***K.tibetana***B***K.dingye* sp. nov. **C***K.lhasa* sp. nov. **D***K.zhui* sp. nov. **E***K.shigatse* sp. nov. **F***K.namling* sp. nov. Scale bars: 0.5 mm.

*Karschiadingye* sp. nov. differs from *K.nubigena* by having fringed flagellum (Fig. 11B), and pedipalpal metatarsus with papillae (Fig. 16D), differs from *K.tibetana* by flagellar complex plumose (*fcp*) setae short (Fig. 11B), from *K.lhasa* sp. nov. and *K.zhui* sp. nov. by cheliceral fixed finger mucron without dorsal crest (Figs 8F, 13F), and from *K.shigatse* sp. nov. and *K.namling* sp. nov. by flagellum without lateral apophysis (Fig. 11B). The female genital operculum is easily recognizable across all known species, its bottom is slightly widened, giving it a trapezoidal appearance (Fig. 17B).

**Figure 12.**
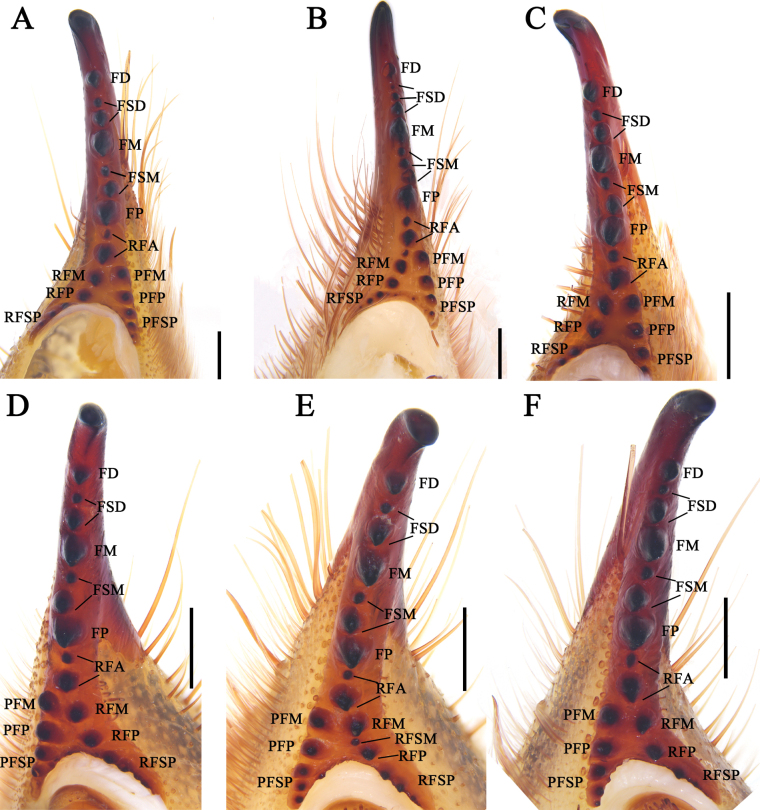
Female fixed finger ventral views **A***K.tibetana***B***K.dingye* sp. nov. **C***K.lhasa* sp. nov. **D***K.zhui* sp. nov. **E***K.shigatse* sp. nov. **F***K.namling* sp. nov. Abbreviations: FD, fixed finger, distal tooth; FSD, fixed finger, subdistal tooth/teeth; FM, fixed finger, medial tooth; FSM, fixed finger, submedial tooth/teeth; FP, fixed finger, proximal tooth; RFA, retrofondal apical tooth/teeth; RFM, retrofondal medial tooth; RFSM, retrofondal submedial tooth/teeth; RFP, retrofondal proximal tooth; RFSP, retrofondal subproximal tooth/teeth; PFM, profondal medial tooth; PFP, profondal proximal tooth; PFSP, profondal subproximal tooth. Scale bars: 0.5 mm.

**Figure 13.**
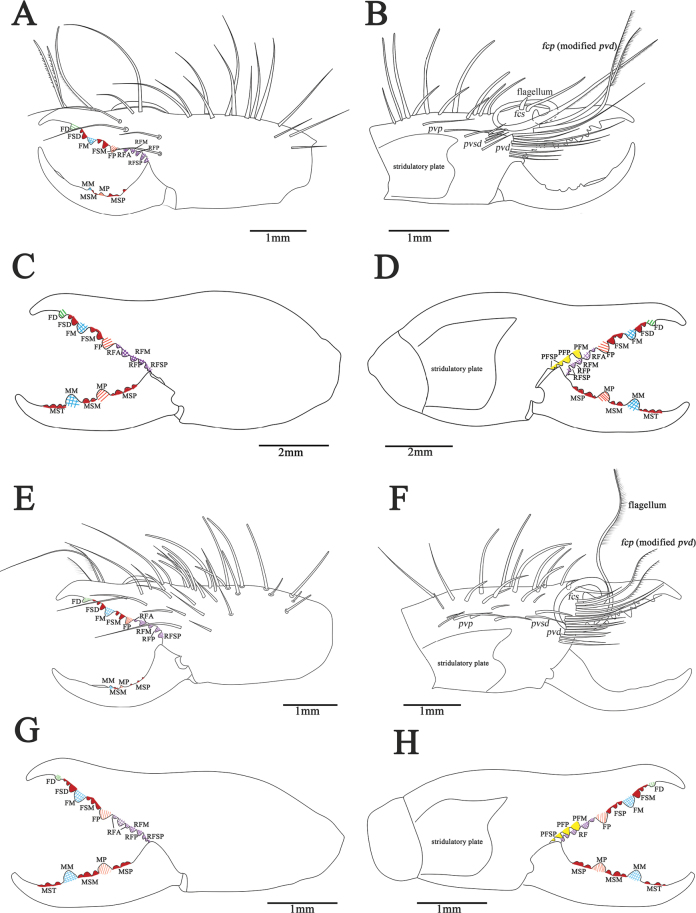
Retrolateral (left) and prolateral (right) cheliceral views **A, B***K.tibetana*, male **C, D***K.tibetana*, female **E, F***K.dingye* sp. nov., holotype male **G, H***K.dingye* sp. nov., paratype female. Abbreviations: FD, fixed finger, distal tooth; FSD, fixed finger, subdistal tooth/teeth; FM, fixed finger, medial tooth; FSM, fixed finger, submedial tooth/teeth; FP, fixed finger, proximal tooth; RF, retrofondal teeth; RFA, retrofondal apical tooth/teeth; RFM, retrofondal medial tooth; RFP, retrofondal proximal tooth; RFSP, retrofondal subproximal tooth/teeth; PF, profondal teeth; PFM, profondal medial tooth; PFP, profondal proximal tooth; PFSP, profondal subproximal tooth; MSP, movable finger, subproximal tooth/teeth; MP, movable finger, proximal tooth; MSM, movable finger, submedial tooth/teeth; MM, movable finger, medial tooth; MST, movable finger, subterminal teeth; *pvd*, proventral distal setae; *fcp* (modified *pvd*), flagellar complex plumose setae; *pdp*, prodorsal proximal setae; *fcs*, flagellar complex subspiniform to spiniform setae; *pvsd*, proventral subdistal setae; *pvd*, proventral distal setae. Scale bars: 1.0 mm (**A, B; E–H**); 2.0 mm (**C, D**).

##### Description.

**Male.** Holotype (MHBU-Sol-XZ2023072701).

***Measurements*.** Total body length 17.58, CL 4.96, CH 1.77, PL 2.11, PW 3.28, A/CP 7.86, CL/CH 2.80. Pedipalp 17.70 (3.68, 5.95, 4.07, 1.51), Leg I 14.65 (3.67, 3.66, 2.72, 1.48, 0.15), Leg II 11.13 (2.74, 2.73, 2.25, 1.03, 0.76), Leg III 14.58 (3.78, 3.77, 2.87, 1.04, 1.16), Leg IV 23.25 (5.45, 6.41, 4.42, 1.38, 1.23).

***Coloration*.** In 95% ethanol-preserved specimens (Fig. 3E, F). The general background brown- yellow. Opisthosoma pale yellow, with black tergites and pale black sternites. Propeltidium tinged with pale brown. Ocular tubercle black. Mesopeltidium and metapeltidium with special black stripes. Chelicerae with manus predominantly yellowish, with some black areas, and a retrolateral view of chelicerae with three black longitudinal stripes (paler than *K.tibetana*). Pedipalps and legs pale yellow, legs III and legs IV tinged with pale brown on distal regions of femora and proximal parts of tibiae. Proximal regions of the pedipalpal femur, tibia, metatarsus, and tarsus tinged with brown. Malleoli white.

***Propeltidium*.** Much wider than long with dense pubescence of thin, short, anteriorly directed setae. Anterior, posterior, and lateral edges with several long, curved spiniform setae, perpendicular to the surface of the propeltidium. Ocular tubercle with one short and four long middle distal spiniform setae which are arranged symmetrically on both sides of the short spiniform setae, one long middle spiniform setae, two short spiniform setae, and numerous shorter, thinner proximal setae (Fig. 6C).

***Chelicerae*.** Fixed finger primary teeth graded as FD < FP < FM. Profondal teeth series with four tiny teeth; retrofondal teeth series with six teeth. Dental formulation of fixed finger: FD-(3)-FM-(2)-FP-(6RF) (4PF). Fixed finger mucron moderately long, without dorsal crest. Movable finger MP tooth about the same size as MM. Dental formulation of movable finger: MM-(2)-MP, with two MSM and three MSP (Figs 8E, 13E). Flagellum, fringed without lateral apophysis, and basal peg expand. The flagellar complex includes two short *fcp* and two short, thick *fcs* (Figs 8F, 11B, 13F). Retrolateral and dorsal surfaces of the manus with large, bifurcated tip setae and short simple tip bristle-like setae; retrolateral and dorsal surfaces of the fixed finger with simple tip setae of different sizes. Retrolateral setose area reaching the FSM teeth; prolateral surface with an array of setal types (Figs 8E, F, 13E, F).

***Opisthosoma*.** The entire surface covered with almost adpressed setae and numerous long, curved, bifurcate setae. Sternite III with two posterior paramedian groups of ctenidia, being gradually larger to posterior. (Fig. 19B); Sternite IV with 17 short peg-like ctenidia extending 1/4 the length of the succeeding sternite (Fig. 19D).

***Pedipalps*.** Entirely covered with short setae and long, thick setae. Tarsus with eleven short, sturdy ventral spines; metatarsus with nine ventral spines not arranged in pairs and with thin papillae (Fig. 16C, D).

***Legs*.** Entirely covered with long, thick setae and short setae. Leg I with no spines and two small claws. Tibias II, III, and IV with a pair of distal spines ventrally. Tibias II and III with a single dorsal spine; metatarsi II and III with a series of three dorsal spines, a pair of distal spines ventrally, and some paired short, thick, spine-shaped bristles over their entire ventral surface. Metatarsus IV also with these paired bristles over its entire ventral surface and two distal spines ventrally.

**Female. Paratype** (MHBU-Sol-XZ2023072701).

***Measurements*.** Total body length 25.64, CL 7.18, CH 2.75, PL 3.23, PW 4.25, A/CP 4.72, CL/CH 2.61, Palp 17.61 (4.16, 4.97, 3.70, 1.24), Leg I 9.84 (2.52, 3.60, 2.57, 1.18, 0.12), Leg II 10.21 (1.48, 2.49, 1.56, 0.83, 0.70), Leg III 12.80 (2.13, 2.87, 2.51, 0.94, 0.10), Leg IV 21.68 (4.06, 5.77, 3.96, 1.13, 1.19).

***Coloration*.** In 95% ethanol-preserved specimens (Fig. 3G, H). Coloration as in the males.

***Propeltidium*.** Much wider than long with a dense pubescence of thin, short, anteriorly directed setae. Anterior, posterior, and lateral edges with several long, curved spiniform setae that perpendicular to the surface of the propeltidium. Ocular tubercle with four middle distal spiniform setae, covered with some long setae and numerous shorter, thinner setae (Fig. 6D).

***Chelicerae*.** Dental formulation of fixed finger: FD-(3)-FM-(3)-FP-(7RF) (4PF). Dental formulation of movable finger: MM-(3)-MP, with three MST (front one tiny) and two MSP. Fondal teeth graded as II, IV, V, tiny I, tiny III, tiny VI, tiny VII retrolaterally; I, II, III, tiny IV prolaterally (Figs 8G, H, 12B, 13G, H).

***Opisthosoma*.** The entire surface covered with almost adpressed setae and numerous long, curved, bifurcate setae. The bottom of the genital operculum slightly widened, resembling a trapezoid (Fig. 17B). Sternite IV with 19 short spine-like ctenidia extending from the edge of sternite IV (Fig. 18B).

***Pedipalps*.** Entirely covered with short setae and long, thick setae and without spines.

***Legs*.** As in the males.

##### Variability.

Males. Total length 14.25–18.76. Body coloration pale yellow to tan. Chelicerae with manus yellowish to brown. The number of cheliceral fixed finger fondal teeth 9–11 (profondal teeth 3–4; retrofondal teeth 6–7). The number of ctenidia on sternite IV 16–18. Pedipalp tarsus with 10–12 spines, metatarsus with 8–10 spines. Females. Total length 20.13–27.68. Variability of body coloration as in males. The number of cheliceral fixed finger fondal teeth 9–12 (profondal teeth 3–5; retrofondal teeth 6–7). MST 2–3, MSP 1–2. The number of ctenidia on sternite IV 17–21. Additionally, we found that all specimen with 3FSD.

##### Distribution and habitat.

China (Xizang). Habitat: meadow (Fig. 2A, B).

##### Remark.

Based on the comparison of genetic distances, with a genetic distance of 0.30% (Table 2) between male and female collected from same locations, we believe that they are same species.

#### ﻿ Karschia (Karschia) lhasa sp. nov.

Taxon classificationAnimaliaSolifugaeKarschiidae

59FC59E6-FA1A-5058-B47E-D6DEA633201E

https://zoobank.org/5F281BD9-03EC-49E6-9A95-1E6E1A096FEF

Figs 1 , 4A–D , 6E, F , 9A–D , 11C , 12C , 14A–D , 16E, F , 17C , 18C , 19E, G , Tables 1 , 2

##### Type material.

***Holotype*** ♂ (MHBU-Sol-XZ2018070501), China: Xizang, Lhasa City, Maizhokunggar County, 29.8268°N, 91.6991°E, ca 3800 m elev., 5. VIII.2018, leg. Yannan Mu. ***Paratypes***: 1♂ (MHBU-Sol-XZ2018070502), 1♀ (MHBU-Sol-XZ2018070503), with same data as holotype.

##### Etymology.

Noun in apposition taken from Lhasa City where this species was collected.

##### Diagnosis.

*Karschialhasa* sp. nov. differs from all *Karschia* species except *K.zhui* sp. nov. by cheliceral fixed finger mucron having dorsal crest (Fig. 11C). *K.lhasa* sp. nov. differs from *K.zhui* sp. nov. by cheliceral fixed finger mucron crescent-shaped dorsal crest broader (Fig. 11C), and pedipalp having more spines and thick papillae (Fig. 16H). The female genital operculum is easily recognizable when compared to that of other species; it has a clear demarcation between the plates. and resembles a fan-shaped structure. (Fig. 17C).

**Figure 14.**
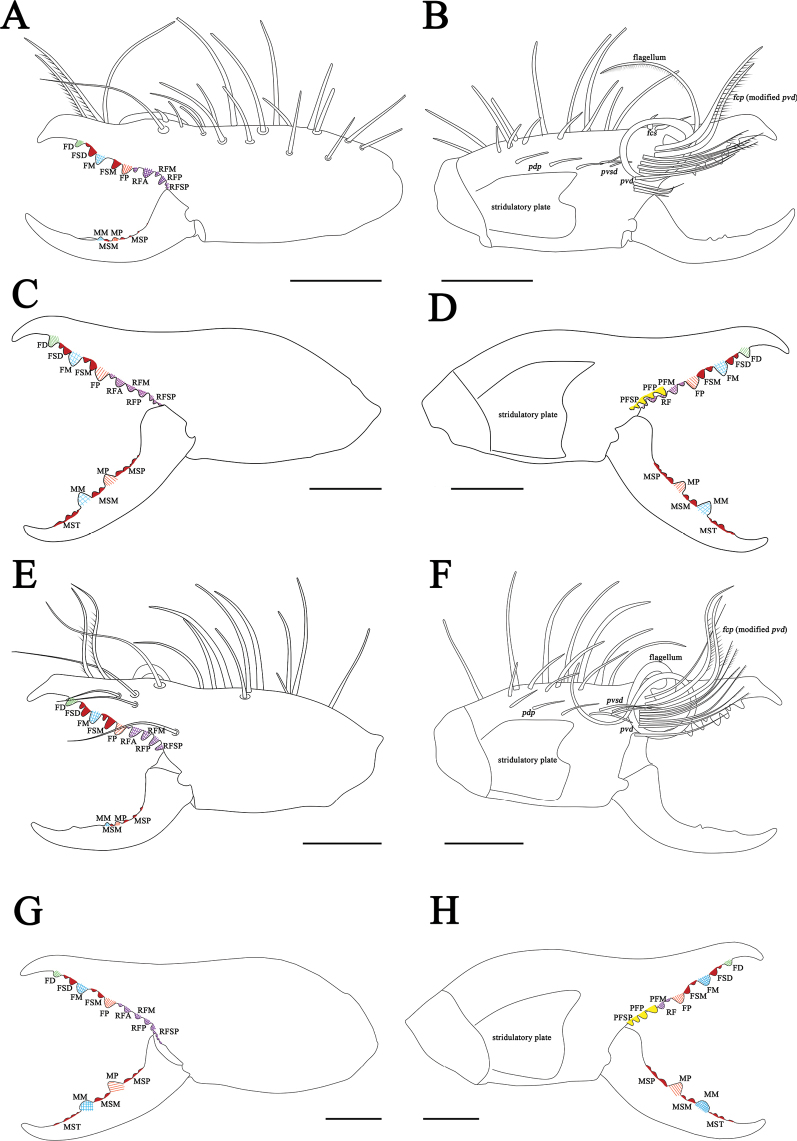
Retrolateral (left) and prolateral (right) cheliceral views **A, B***K.lhasa* sp. nov., holotype male **C, D***K.lhasa* sp. nov., paratype female **E, F***K.zhui* sp. nov., holotype male **G, H***K.zhui* sp. nov., paratype female. Abbreviations: FD, fixed finger, distal tooth; FSD, fixed finger, subdistal tooth/teeth; FM, fixed finger, medial tooth; FSM, fixed finger, submedial tooth/teeth; FP, fixed finger, proximal tooth; RF, retrofondal teeth; RFA, retrofondal apical tooth/teeth; RFM, retrofondal medial tooth; RFP, retrofondal proximal tooth; RFSP, retrofondal subproximal tooth/teeth; PF, profondal teeth; PFM, profondal medial tooth; PFP, profondal proximal tooth; PFSP, profondal subproximal tooth; MSP, movable finger, subproximal tooth/teeth; MP, movable finger, proximal tooth; MSM, movable finger, submedial tooth/teeth; MM, movable finger, medial tooth; MST, movable finger, subterminal teeth; *pvd*, proventral distal setae; *fcp* (modified *pvd*), flagellar complex plumose setae; *pdp*, prodorsal proximal setae; *fcs*, flagellar complex subspiniform to spiniform setae; *pvsd*, proventral subdistal setae; *pvd*, proventral distal setae. Scale bars: 1.0 mm.

**Figure 15.**
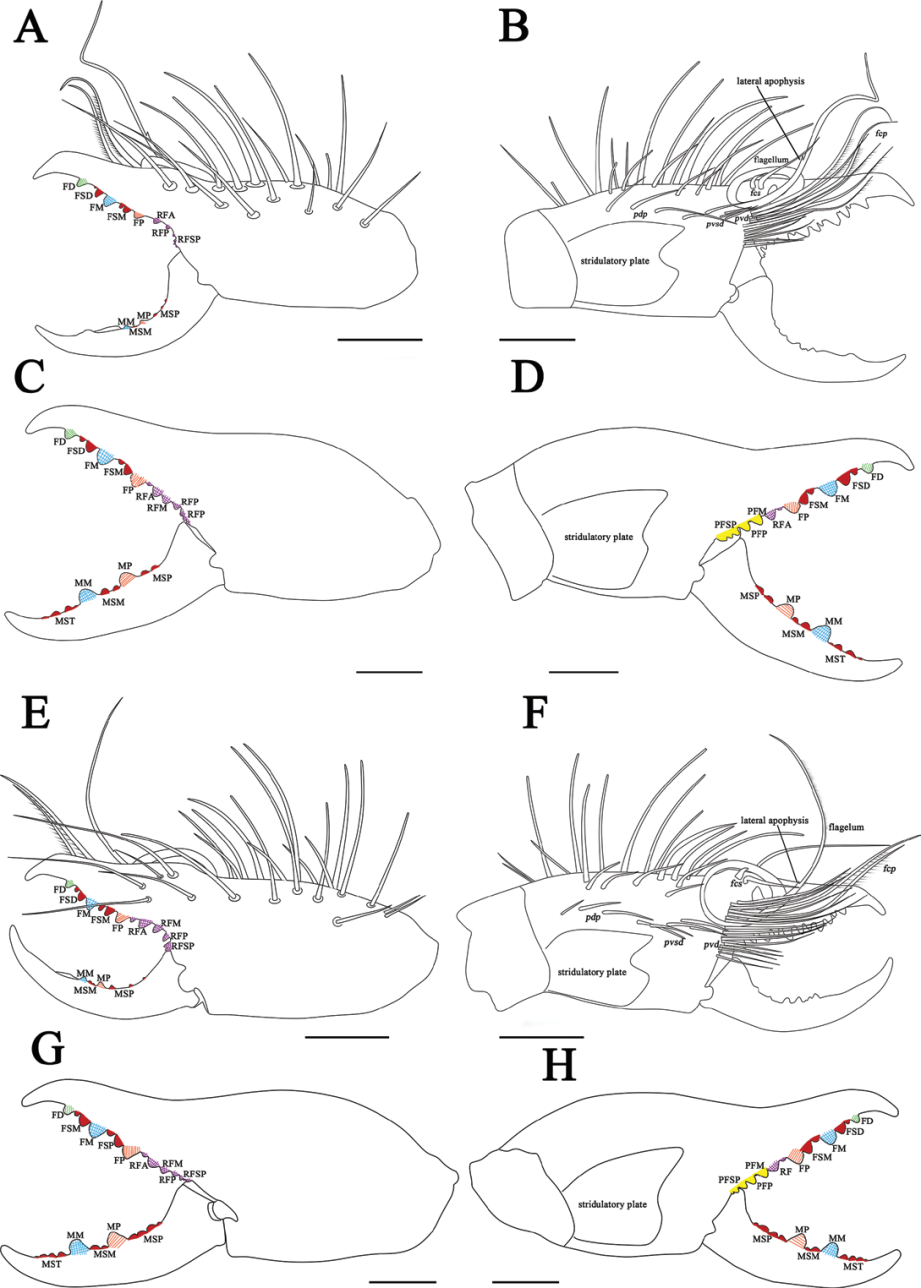
Retrolateral (left) and prolateral (right) cheliceral views **A, B***K.shigatse* sp. nov., holotype male **C, D***K.shigatse* sp. nov., paratype female **E, F***K.namling* sp. nov., holotype male **G, H***K.namling* sp. nov., paratype female. Abbreviations: FD, fixed finger, distal tooth; FSD, fixed finger, subdistal tooth/teeth; FM, fixed finger, medial tooth; FSM, fixed finger, submedial tooth/teeth; FP, fixed finger, proximal tooth; RF, retrofondal teeth; RFA, retrofondal apical tooth/teeth; RFM, retrofondal medial tooth; RFP, retrofondal proximal tooth; RFSP, retrofondal subproximal tooth/teeth; PF, profondal teeth; PFM, profondal medial tooth; PFP, profondal proximal tooth; PFSP, profondal subproximal tooth; MSP, movable finger, subproximal tooth/teeth; MP, movable finger, proximal tooth; MSM, movable finger, submedial tooth/teeth; MM, movable finger, medial tooth; MST, movable finger, subterminal teeth; *pvd*, proventral distal setae; *fcp* (modified *pvd*), flagellar complex plumose setae; *pdp*, prodo rsal proximal setae; *fcs*, flagellar complex subspiniform to spiniform setae; *pvsd*, proventral subdistal setae; *pvd*, proventral distal setae. Scale bars: 1.0 mm.

##### Description.

**Male** Holotype (MHBU-Sol-XZ2018070501).

***Measurements*.** Total body length 16.60, CL 4.03, CH 1.26, PL 2.01, PW 2.59, A/CP 7.57, CL/CH 3.19. Pedipalp 15.64 (3.12, 4.69, 2.46, 1.04), Leg I 10.06 (2.18, 2.78, 2.00, 0.97, 0.18), Leg II 9.28 (1.37, 2.16, 1.69, 0.74, 0.45), Leg III 12.95 (2.13, 3.13, 2.49, 0.71, 0.78), Leg IV 19.99 (4.76, 5.35, 3.75, 1.29, 1.28).

***Coloration*.** In 75% ethanol-preserved specimens (Fig. 4A, B). The general background pale yellow. Opisthosoma grey-yellow, with black tergites and pale black sternites. Propeltidium tinged pale brown. Ocular tubercle black. Mesopeltidium and metapeltidium with special black stripes. Chelicerae with manus predominantly brown-yellow, with some black areas, and a retrolateral view of chelicerae with three black longitudinal stripes. Pedipalps and legs yellow, legs III and legs IV tinged with pale brown on distal regions of femora and proximal parts of tibiae. Proximal regions of the pedipalpal femur, tibia, metatarsus, and tarsus tinged with brown. Malleoli yellow.

***Propeltidium*.** Slightly wider than long with a dense pubescence of thin, short, anteriorly directed setae. Anterior, posterior, and lateral edges with several long, curved spiniform setae that perpendicular to the surface of the propeltidium. Ocular tubercle with one short and four long middle distal spiniform setae, one long median spiniform setae, two shorter posterior spiniform setae, and numerous short, thin posterior setae (Fig. 6E).

***Chelicerae*.** Fixed finger primary teeth graded as FD < FP≈FM. Profondal teeth series with three or four tiny teeth; retrofondal teeth series with six teeth. Dental formulation of fixed finger: FD-(2)-FM-(1)-FP-(6RF) (3PF). Fixed finger mucron with wider and crescent-shaped dorsal crest. Movable finger MP tooth about the same size as MM. Dental formulation of movable finger: MM-(2)-MP, with one tiny MSM and four MSP (Figs 9A, B, 14A, B). Flagellum coiled, fringed and sessile, without lateral apophysis. The flagellar complex includes two medium length *fcp* and two short, thick *fcs* (Figs 9B, 11C, 14B). Retrolateral and dorsal surfaces of the manus with large, bifurcated tip setae and short, simple tip bristle-like setae; retrolateral and dorsal surfaces of the fixed finger with simple tip setae of different sizes. Retrolateral setose area reaching the FSM teeth; prolateral surface with an array of setal types (Figs 9A, B, 14A, B).

***Opisthosoma*.** The entire surface covered with almost adpressed setae and numerous long, curved, bifurcate setae. Sternite III with short, thin ctenidia: 14+15 (on the right and left side, respectively) (Fig. 19E); Sternite IV with 13 short peg-like ctenidia, the length of which almost 1/3 the width of the succeeding sternite (Fig. 19G).

**Figure 16.**
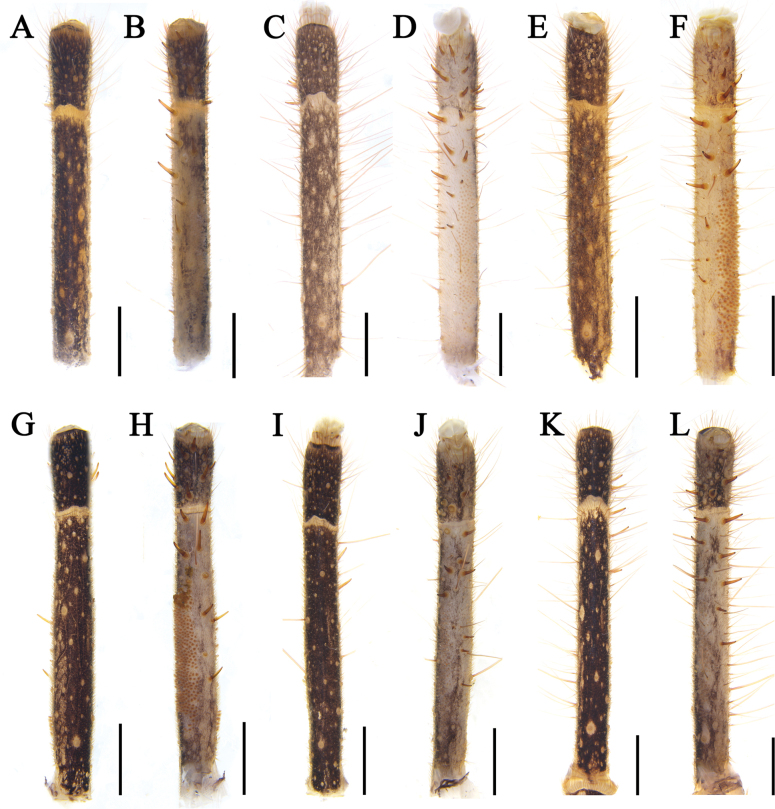
Male pedipalp **A, B***K.tibetana***C, D***K.dingye* sp. nov. **E, F***K.lhasa* sp. nov. **G, H***K.zhui* sp. nov. **I, J***K.shigatse* sp. nov. **K, L***K.namling* sp. nov. Scale bars: 1.0 mm.

***Pedipalps*.** Totally covered with short setae and long, thick setae. Tarsus not swollen with five sturdy ventral spines; metatarsus with eight ventral spines not arranged in pairs and with thick papillae (Fig. 16E, F).

***Legs*.** Totally covered with long, thick setae and short setae. Leg I with no spines and two small claws. Tibias II, III, and IV with a pair of distal spines ventrally. Tibias II and III with a single dorsal spine. Metatarsi II and III with a series of three dorsal spines, a pair of distal spines ventrally, and some paired short, thick, spine-shaped bristles over their entire ventral surface; metatarsus IV also with these paired bristles over its entire ventral surface and two distal spines ventrally.

**Female. Paratype** (MHBU-Sol-XZ2018070503).

***Measurements*.** Total body length 16.69, CL 5.10, CH 1.76, PL 2.45, PW 3.53, A/CP 4.60, CL/CH 2.90. Pedipalp 12.55 (3.13, 3.52, 2.73, 1.05), Leg I 8.15 (1.60, 2.32, 1.70, 0.93, 0.14), Leg II 7.66 (1.05, 1.53, 1.38, 0.98, 0.61), Leg III 7.74 (0.81, 1.71, 2.23, 0.98), Leg IV 14.06 (1.96, 4.25, 2.23, 0.74, 0.78).

***Coloration*.** In 75% ethanol-preserved specimens (Fig. 4C, D). Coloration as in the males. *Propeltidium*. Much wider than long with a dense pubescence of thin, short, anteriorly directed setae. Anterior, posterior, and lateral edges with several long, curved spiniform setae that perpendicular to the surface of the propeltidium. Ocular tubercle with four middle distal spiniform setae, two middle spiniform setae, and two posterior spiniform setae (Fig. 6F).

***Chelicerae*.** Dental formulation of fixed finger: FD-(2)-FM-(2)-FP-(6RF) (4PF). Dental formulation of movable finger: MM-(2)-MP, with four MST and five MSP. Fondal teeth graded as II, III, IV, I, V, VI retrolaterally; I, II, III, IV prolaterally (Figs 9C, D, 12C, 14C, D).

**Figure 17.**
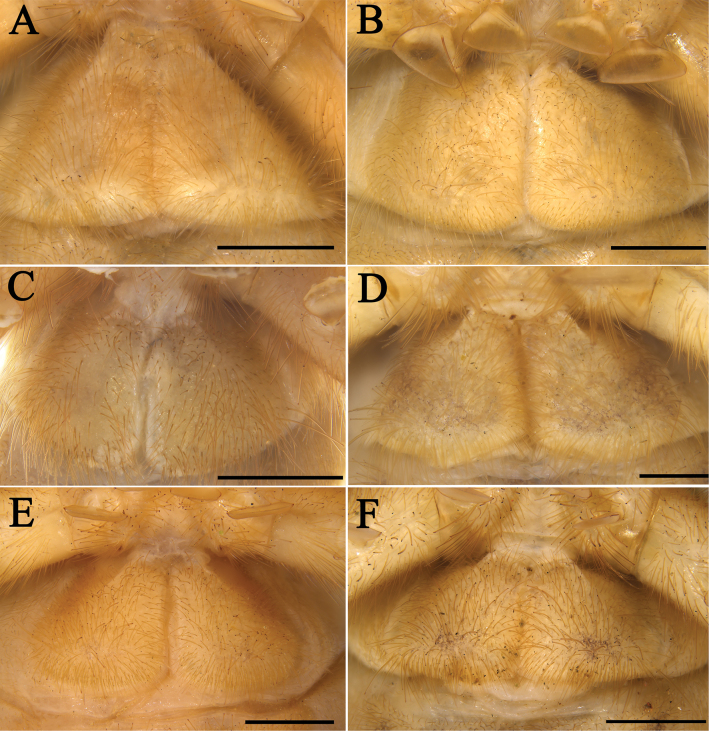
Genital operculum of female **A***K.tibetana***B***K.dingye* sp. nov. **C***K.lhasa* sp. nov. **D***K.zhui* sp. nov. **E***K.shigatse* sp. nov. **F***K.namling* sp. nov. Scale bars: 1.0 mm.

**Figure 18.**
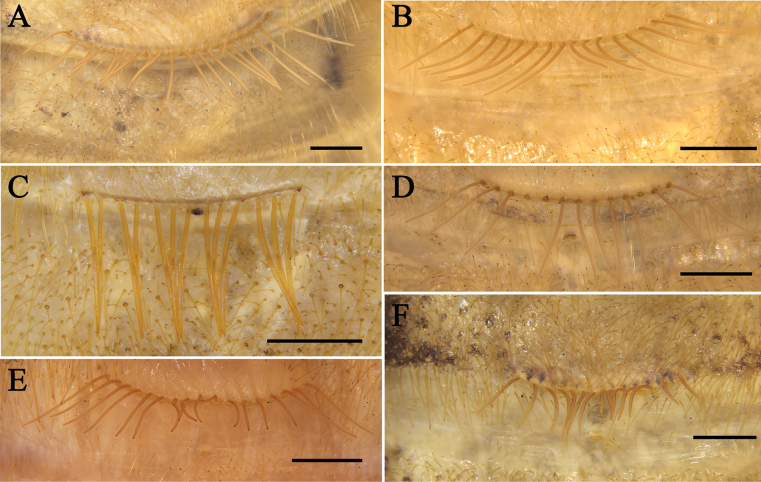
Ctenidia on sternite IV of female **A***K.tibetana***B***K.dingye* sp. nov. **C***K.lhasa* sp. nov. **D***K.zhui* sp. nov. **E***K.shigatse* sp. nov. **F***K.namling* sp. nov. Scale bars: 0.5 mm.

***Opisthosoma*.** The entire surface covered with almost adpressed setae and numerous long, curved, bifurcate setae. The bottom of the genital operculum slightly widened, resembling a fan-shaped structure (with chitinized folds) between and behind it (Fig. 17C). Sternite IV with 13 long needle-like ctenidia extending 3/4 the length of the succeeding sternite (Fig. 18C).

***Pedipalps*.** Totally covered with short setae and long, thick setae without spines.

***Legs*.** As in the males.

##### Variability.

Males. Total length 15.52–16.60. The number of cheliceral fixed finger fondal teeth 9–10 (profondal teeth 3–4). The number of ctenidia on sternite III 28–32 and on sternite IV 13–14. Pedipalp tarsus with 5–6 spines, metatarsus with 8–10 spines.

##### Distribution and habitat.

China (Xizang). Habitat: wild grassy slope (Fig. 2C).

##### Remark.

Based on the comparison of genetic distances, with a genetic distance of 0% (Table 2) between male and female collected from same locations, we believe that they are same species.

#### ﻿ Karschia (Karschia) zhui sp. nov.

Taxon classificationAnimaliaSolifugaeKarschiidae

678B85C3-5C89-53CC-83D6-822353322E2D

https://zoobank.org/CC55BE2C-C5C7-4B7D-A4A8-8019022A4339

Figs 1 , 4E–H , 7A, B , 9E–H , 11D , 12D , 14E–H , 16G, H , 17D , 18D , 19F, H , Tables 1 , 2

##### Type material.

***Holotype*** ♂ (MHBU-Sol-XZ2022070401), China: Xizang, Lhasa City, Drepung Monastery, 29.6697°N, 91.0548°E, 3672.7 m elev., 4.VII.2022, leg. Wenlong Fan. ***Paratype***: 1♀ (MHBU-Sol-XZ2023070501), China: Xizang, Lhasa City, Drepung Monastery, 29.6758°N, 91.0490°E, 3903 m elev., 5.VII.2023, leg. Quanyu Ji.

##### Etymology.

Patronym honors Prof. Ming-Sheng Zhu (Hebei University), who significantly contributed to arachnological studies in China.

##### Diagnosis.

*Karschiazhui* sp. nov. differs from all *Karschia* species except *K.lhasa* sp. nov. by cheliceral fixed finger mucron having dorsal crest (Figs 9F, 11D). *K.zhui* sp. nov. differs from *K.lhasa* sp. nov. by cheliceral fixed finger mucron dorsal crest small crescent-shaped (Figs 9F, 11D), and pedipalp having less spines and thin papillae (Fig. 16H). Female genital operculum like *K.tibetana*, but can be diagnosed by the lower edge, which is somewhat convex, not flat (Fig. 17D).

##### Description.

**Male.** Holotype (MHBU-Sol-XZ2022070401).

***Measurements*.** Total body length 14.10, CL 4.33, CH 1.61, PL 2.11, PW 2.96, A/CP 7.50, CL/CH 2.69. Pedipalp 16.39 (3.83, 5.41, 3.66, 1.33), Leg I 11.95 (3.06, 3.32, 2.41, 0.97, 0.14), Leg II 10.08 (1.73, 2.33, 1.77, 0.63, 0.67), Leg III 12.31 (2.57, 3.11, 1.56, 0.52, 0.61), Leg IV 19.95 (3.88, 5.36, 2.94, 1.19, 0.98).

***Coloration*.** In 95% ethanol-preserved specimens (Fig. 4E, F). The general background pale yellow. Opisthosoma brow yellow, with black tergites and pale black sternites. Propeltidium black tinged with pale brown. Ocular tubercle black. Mesopeltidium and metapeltidium with special black stripes. Chelicerae with manus predominantly yellowish, with some black areas, and a retrolateral view of chelicerae with three black longitudinal stripes. Pedipalps and legs pale brown-yellow, legs III and legs IV tinged with pale brown on distal regions of femora and proximal parts of tibiae. Proximal regions of the pedipalpal femur, tibia, metatarsus, and tarsus tinged with brown. Malleoli white.

***Propeltidium*.** Wider than long with a dense pubescence of thin, short, anteriorly directed setae. Anterior, posterior, and lateral edges with several long, curved spiniform setae that perpendicular to the surface of the propeltidium. Ocular tubercle with four middle distal spiniform setae, one middle spiniform setae, and two proximal spiniform setae (Fig. 7A).

***Chelicerae*.** Fixed finger primary teeth graded as FP < FM ≈ FD. Profondal teeth series with four or five tiny teeth; retrofondal teeth series with six teeth. Dental formulation of fixed finger: FD-(2)-FM-(2)-FP-(6RF) (4PF). Fixed finger mucron with crescent-shaped dorsal crest smaller than *K.lhasa*. Movable finger MP tooth about the same size as MM. Dental formulation of movable finger: MM-(1)-MP, with one tiny MSM and three MSP (Figs 9E, 14E). Flagellum coiled, fringed and sessile, without lateral apophysis. The flagellar complex includes two medium length *fcp* and two short, thick *fcs*. (Figs 9E, 11D, 14F). Retrolateral and dorsal surfaces of the manus with large, bifurcated tip setae and short, simple tip bristle-like setae; retrolateral and dorsal surfaces of the fixed finger with simple tip setae of different sizes. Retrolateral setose area reaching the FSM teeth; prolateral surface with an array of setal types (Figs 9E, F, 14E, F).

**Figure 19.**
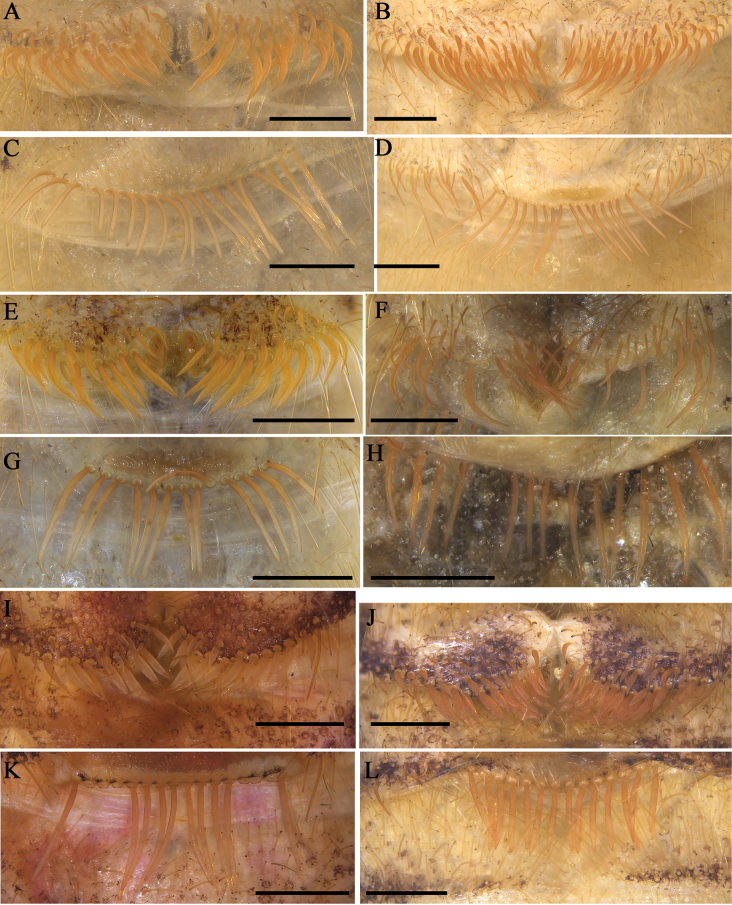
Ctenidia on sternite III (A, B, E, F, I, J) and sternite IV (C, D, G, H, K, L) of male **A, C***K.tibetana***B, D***K.dingye* sp. nov. **E, G***K.lhasa* sp. nov. **F, H***K.zhui* sp. nov. **I, K.***K.shigatse* sp. nov. **J, L***K.namling* sp. nov. Scale bars: 0.5 mm.

***Opisthosoma*.** The entire surface covered with almost adpressed setae and numerous long, curved, bifurcate setae. Sternite III with a row of disordered ctenidia (Fig. 19F). Sternite IV with 16 long peg-like ctenidia, the length of which almost equal to 1/2 the width of the succeeding sternite (Fig. 19H).

***Pedipalps*.** Totally covered with short setae and long, thick setae. Tarsus with nine sturdy ventral spines; metatarsus with 11 ventral spines not arranged in pairs and with thick papillae (Fig. 16G, H).

***Legs*.** Totally covered with long, thick setae and short setae. Leg I with no spines and two small claws. Tibias II, III, and IV with a pair of distal spines ventrally. Tibias II and III with a single dorsal spine; metatarsi II and III with a series of three dorsal spines, a pair of distal spines ventrally, and some paired short, thick, spine-shaped bristles over their entire ventral surface. Metatarsus IV also with these paired bristles over its entire ventral surface and two distal spines ventrally.

**Female. Paratype** (MHBU-Sol-XZ2023070501).

***Measurements*.** Total body length 21.52, CL 6.81, CH 2.50, PL 2.94, PW 4.27, A/CP 4.47, CL/CH 2.72. Pedipalp 13.39 (2.74, 4.28, 3.31, 1.11), Leg IV 11.35 (1.98, 3.13, 2.21, 1.09, 0.11), Leg II 10.29 (1.53, 2.13, 1.62, 0.87, 0.79), Leg III 12.78 (1.69, 2.70, 2.06, 0.62, 0.96), Leg IV 18.81 (4.41, 4.29, 2.25, 1.05, 1.19).

***Coloration*.** In 75% ethanol-preserved specimens (Fig. 4G, H). Coloration as in the males.

***Propeltidium*.** Much wider than long with a dense pubescence of thin, short, anteriorly directed setae. Anterior, posterior, and lateral edges with several long, curved spiniform setae that stand perpendicular to the surface of the propeltidium. Ocular tubercle with four middle distal spiniform setae, one middle spiniform setae, and two proximal spiniform setae (Fig. 7B).

***Chelicerae*.** Dental formulation of fixed finger: FD-(2)-FM-(2)-FP-(8RF) (4PF). Dental formulation of movable finger: MM-(2)-MP, with four MST and three MSP. Fondal teeth graded as II, III, IV, I, V, VI, tiny VII, tiny VIII retrolaterally; II, I, III, tiny IV prolaterally (Figs 9G, H, 12D, 14G, H).

***Opisthosoma*.** The entire surface covered with almost adpressed setae and numerous long, curved, bifurcate setae. The bottom of the genital operculum slightly widened, resembling a triangular-shape (with chitinized folds) between and behind them (Fig. 17D). Sternite IV with 13 long needle-like ctenidia extending one-third the length of the succeeding sternite (Fig. 18D).

***Pedipalps*.** Totally covered with short and long setae, thick setae and without spines.

***Legs*.** As in the males.

##### Distribution and habitat.

China (Xizang). Habitat: shrubbery (Fig. 2D).

##### Remark.

Based on the comparison of genetic distances, with a genetic distance of 0.15% (Table 2) between the male and female collected from the same location, we believe that they are same species.

#### ﻿ Karschia (Karschia) shigatse sp. nov.

Taxon classificationAnimaliaSolifugaeKarschiidae

8941120D-5CE0-5476-B2D7-9AAA196D6B3F

https://zoobank.org/A84E6F7D-8AC8-45D1-A756-F0172B2C3289

Figs 1 , 5A–D , 7C, D , 10A–D , 11E , 12E , 15A–D , 16I, J , 17E , 18E , 19I, K , Tables 1 , 2

##### Type material.

***Holotype*** ♂ (MHBU-Sol-XZ2022071501), China: Xizang, Shigatse Prefecture, Nyalam County, Mainqu Town, 28.6773°N, 86.1395°E, 4552.71 m elev., 15.VII.2022, leg. Wenlong Fan. ***Paratype***: 1♀ (MHBU-Sol-XZ2023072101), China: Xizang, Shigatse Prefecture Gyirong County, Zheba Town, 29.1976°N, 85.3571°E, 4605.8 m elev., 21.VII.2023, leg. Xiangbo Guo.

##### Etymology.

Noun in apposition taken from Shigatse Prefecture where this species was collected.

##### Diagnosis.

*Karschiashigatse* sp. nov. differs from *K.nubigena* by having fringed flagellum (Fig. 11E), from *K.tibetana*, *K.dingye* sp. nov., *K.lhasa* sp. nov. and *K.zhui* sp. nov. by flagellum with lateral apophysis (Fig. 11E), and from *K.namling* sp. nov. by wide cheliceral fixed finger mucron, and lateral apophysis of flagellum larger (Fig. 11E). Female differs from other species by genital operculum equilateral subtriangular and with no clear demarcation between the plates. (Fig. 17E).

##### Description.

**Male.** Holotype (MHBU-Sol-XZ2022071501).

***Measurements*.** Total body length 15.52, CL 4.88, CH 1.56, PL 2.26, PW 2.74, A/CP 7.24, CL/CH 3.14. Pedipalp 19.38 (5.35, 5.62, 4.12, 1.26), Leg I 13.51 (3.17, 3.90, 2.23, 1.02, 0.19), Leg II 10.95 (2.66, 2.33, 1.37, 0.92, 0.70), Leg III 16.87 (3.62, 4.59, 3.02, 0.54, 0.87), Leg IV 18.77 (4.71, 6.49, 4.71, 1.57, 1.31).

***Coloration*.** In 95% ethanol-preserved specimens (Fig. 5A, B). The general background pale yellowish. The opisthosoma slightly darker, with black tergites and yellow around the black sternites. Propeltidium pale tan and tinged with pale brown. Ocular tubercle black. Mesopeltidium and metapeltidium with special black stripes. Chelicerae with manus predominantly yellowish, with some black areas, and a retrolateral view of chelicerae with three black longitudinal stripes. Pedipalps and legs pale yellow, legs III and legs IV tinged with pale brown on distal regions of femora and proximal parts of tibiae. Proximal regions of the pedipalpal femur, tibia, metatarsus, and tarsus tinged with brown. Malleoli yellow.

***Propeltidium*.** Wider than long with a dense pubescence of thin, short, anteriorly directed setae. Anterior, posterior, and lateral edges with several long, curved spiniform setae that perpendicular to the surface of the propeltidium. Ocular tubercle with two middle distal spiniform setae and one middle spiniform setae (Fig. 7C). *Chelicerae*. Fixed finger primary teeth graded as FP < FD < FM. Profondal teeth series with three tiny teeth; retrofondal teeth series with seven teeth. Dental formulation of fixed finger: FD-(2)-FM-(2)-FP-(7RF) (3PF). Fixed finger mucron without dorsal crest. Movable finger MP tooth about the same size as MM. Dental formulation of movable finger: MM-(2)-MP, with two tiny MSM and three MSP (Figs 10A, 15A). Flagellum coiled, fringed and sessile, with lateral apophysis. The flagellar complex includes two medium length *fcp* and two short, thick *fcs* (Figs 10B, 11E, 15B). Retrolateral and dorsal surfaces of the manus with large, bifurcated tip setae and short, simple tip bristle-like setae; retrolateral and dorsal surfaces of the fixed finger with simple tip setae of different sizes. Retrolateral setose area reaching the FSM teeth; prolateral surface with an array of setal types (Figs 10A, B, 15A, B).

***Opisthosoma*.** Entire surface covered almost adpressed setae, and numerous long, curved, bifurcate setae. Sternite III with 21 pine needle-like ctenidia (Fig. 19I). Sternite IV with 15 long peg-like ctenidia, the length of which almost equal to half the width of the succeeding sternite (Fig. 19K).

***Pedipalps*.** Totally covered with short setae and long, thick setae. Tarsus with eight sturdy ventral spines; metatarsus with 10 ventral spines not arranged in pairs and with thin papillae (Fig. 16I, J).

***Legs*.** Totally covered with long, thick setae and short setae. Leg I with no spines and two small claws. Tibias II, III, and IV with a pair of distal spines ventrally. Tibias II and III with a single dorsal spine; metatarsi II and III with a series of three dorsal spines, a pair of distal spines ventrally, and some paired short, thick, spine-shaped bristles over their entire ventral surface. Metatarsus IV also with these paired bristles over its entire ventral surface and two distal spines ventrally.

**Female. Paratype** (MHBU-Sol-XZ2023072101).

***Measurements*.** Total body length 24.17, CL 6.84, CH 2.47, PL 2.63, PW 3.73, A/CP 4.97, CL/CH 2.77. Pedipalp 16.57 (4.08, 4.48, 3.50, 1.24), Leg I 12.72 (3.69, 3.37, 2.04, 0.97, 0.19), Leg II 10.86 (1.86, 2.41, 1.91, 0.75, 0.61), Leg III 13.77 (2.44, 3.29, 2.24, 0.72, 0.83), Leg IV 17.75 (4.07, 4.08, 2.61, 1.44, 0.78).

***Coloration*.** In 75% ethanol-preserved specimens (Fig. 5C, D). Coloration as in the males.

***Propeltidium*.** Much wider than long with a dense pubescence of thin, short, anteriorly directed setae. Anterior, posterior, and lateral edges with several long, curved spiniform setae that perpendicular to the surface of the propeltidium. Ocular tubercle with four middle distal spiniform setae and three middle spiniform setae arranged in a triangle shape (Fig. 7E).

***Chelicerae*.** Dental formulation of fixed finger: FD-(2)-FM-(2)-FP-(8RF) (5PF). Dental formulation of movable finger: MM-(2)-MP, with four MST and two MSP. Fondal teeth graded as II, III, V, VI, VII, I, IV, tiny VIII retrolaterally; I, II, III, IV, V prolaterally (Figs 10C, D, 12E, 15C, D).

***Opisthosoma*.** The entire surface covered with almost adpressed setae and numerous long, curved, bifurcate setae. Genital operculum equilateral subtriangular and with no clear demarcation between the plates. The rear edge of the genital sternite not chitinized (Fig. 17E). Sternite IV with 17 long needle-like ctenidia extending a half of the length of the succeeding sternite (Fig. 18E).

***Pedipalps***. Totally covered with short setae and long, thick setae and without spines.

***Legs*.** As in the males.

##### Distribution and habitat.

China (Xizang). Habitat: desert grassland (Fig. 2E–G).

##### Remark.

Based on the comparison of genetic distances, with a genetic distance of 2.20% (Table 2) between male and female collected from different near locations, we believe that they are same species.

#### ﻿ Karschia (Karschia) namling sp. nov.

Taxon classificationAnimaliaSolifugaeKarschiidae

B6E20436-9118-55ED-8C02-2D2A376881F0

https://zoobank.org/D73D6B1F-73C4-4D8A-8802-AF0C93B46AE6

Figs 1 , 2H , 5E–H , 7E, F , 10E–H , 11F , 12F , 15E–H , 16K, L , 17F , 18E , 19J, L , Tables 1 , 2

##### Type material.

***Holotype*** ♂ (MHBU-Sol-XZ2023073001), China: Xizang, Namling County, Nubma Town, 29.5172°N, 89.6237°E, 4016.27 m elev., 30. VIII.2023, leg. Yanmeng Hou, Zhiyong Yang. ***Paratypes***: 1♂ (MHBU-Sol-XZ2023073002), 4♀♀ (MHBU-Sol-XZ2023073003–07), same data as holotype.

##### Etymology.

Noun in apposition taken from Namling County, where this species was collected.

##### Diagnosis.

*K.namling* sp. nov. differs from *K.nubigena* by have fringed flagellum (Fig. 11F), differs from *K.tibetana*, *K.dingye* sp. nov., *K.lhasa* sp. nov. and *K.zhui* sp. nov. by flagellum with lateral apophysis (Fig. 11F), and from *Karschiashigatse* sp. nov. by less wide cheliceral fixed finger mucron, flagellum with small lateral apophysis (Fig. 11F). Female differs from other species by its genital operculum triangular (Fig. 17F) and ctenidia on sternite IV very short (Fig. 18F).

##### Description.

**Male.** Holotype (MHBU-Sol-XZ2023073001).

***Measurements*.** Total body length 16.61, CL 4.68, CH 1.58, PL 2.35, PW 3.04, A/CP 8.66, CL/CH 2.97. Pedipalp 22.21 (6.51, 6.56, 4.60, 1.50), Leg I 14.99 (3.56, 3.93, 2.59, 1.36, 0.12), Leg II 11.46 (2.52, 2.65, 1.46, 1.17, 0.19), Leg III 16.72 (3.45, 4.39, 2.86, 0.92, 0.92), Leg IV 23.73 (4.81, 6.47, 3.54, 1.42, 1.43).

***Coloration*.** In 95% ethanol-preserved specimens (Fig. 5E, F). The general background pale yellowish. Opisthosoma slightly darker, with black tergites and yellow around the black sternites. Propeltidium pale tan and tinged with pale brown. Ocular tubercle black. Mesopeltidium and metapeltidium with special black stripes. Chelicerae with manus predominantly brown yellowish, with some black areas, and a retrolateral view of chelicerae with three black longitudinal stripes. Pedipalps and legs pale yellow, legs III and legs IV tinged with pale brown on distal regions of femora and proximal parts of tibiae. Proximal regions of the pedipalpal femur, tibia, metatarsus, and tarsus were tinged with brown. Malleoli white.

***Propeltidium*.** Wider than long with a dense pubescence of thin, short, anteriorly directed setae. Anterior, posterior, and lateral edges with several long, curved spiniform setae that stand perpendicular to the surface of the propeltidium. Ocular tubercle with four middle distal spiniform setae, one middle spiniform setae, and one proximal spiniform setae. (Fig. 7E).

***Chelicerae*.** Fixed finger primary teeth graded as FD < FM ≈ FP. Profondal teeth series with three tiny teeth; retrofondal teeth series with six teeth. Dental formulation of fixed finger: FD-(2)-FM-(2)-FP-(6RF) (3PF). Fixed finger mucron without dorsal crest. Movable finger MP tooth about the same size as MM. Dental formulation of movable finger: MM-(2)-MP, with two tiny MSM and three MSP (Figs 10E, 15E). Flagellum coiled, fringed and sessile, with a small lateral apophysis. The flagellar complex includes two short *fcp* and two short, thick *fcs* (Figs 10F, 11F, 15F). Retrolateral and dorsal surfaces of the manus with large, bifurcated tip setae and short, simple tip bristle-like setae; retrolateral and dorsal surfaces of the fixed finger with simple tip setae of different sizes. Retrolateral setose area reaching the FSM teeth; prolateral surface with an array of setal types (Figs 10E, F, 15E, F).

***Opisthosoma*.** The entire surface covered with almost adpressed setae and numerous long, curved, bifurcate setae. Sternite III with numbers short and cylindrical ctenidia (Fig. 19J). Sternite IV with 14 long peg-like ctenidia, the length of which almost 1/3 the width of the succeeding sternite (Fig. 19L).

***Pedipalps*.** Totally covered with short setae and long, thick setae. Tarsus with six sturdy ventral spines; metatarsus with eight ventral spines not arranged in pairs and with thick papillae (Fig. 16K, L).

***Legs*.** Totally covered with long, thick setae and short setae. Leg I with no spines and two small claws. Tibias II, III, and IV with a pair of distal spines ventrally. Tibias II and III with a single dorsal spine; metatarsi II and III with a series of three dorsal spines, a pair of distal spines ventrally, and some paired short, thick, spine-shaped bristles over their entire ventral surface. Metatarsus IV also with these paired bristles over its entire ventral surface and two distal spines ventrally.

**Female. Paratype.** (MHBU-Sol-XZ2023073003).

***Measurements*.** Total body length 21.28, CL 6.77, CH 2.53, PL 2.76, PW 4.39, A/CP 4.6, CL/CH 2.67. Pedipalp 16.16 (4.70, 4.40, 3.63, 1.25), Leg I 9.872 (2.18, 2.89, 1.66, 0.98, 0.19), Leg II 10.49 (1.93, 2.21, 1.80, 0.84, 0.66), Leg III 11.56 (2.04, 2.96, 1.44, 0.43, 0.50), Leg IV 17.90 (4.06, 4.74, 2.06, 0.49, 0.99).

***Coloration*.** In 75% ethanol-preserved specimens (Fig. 5G, H). Coloration as in the males.

***Propeltidium*.** Much wider than long with a dense pubescence of thin, short, anteriorly directed setae. Anterior, posterior, and lateral edges with several long, curved spiniform setae that perpendicular to the surface of the propeltidium. Ocular tubercle with four middle distal spiniform setae and three middle spiniform setae arranged in a triangle shape (Fig. 7F).

***Chelicerae*.** Dental formulation of fixed finger: FD-(2)-FM-(2)-FP-(6RF) (5PF). Dental formulation of movable finger: MM-(2)-MP, with four MST and four MSP. Fondal teeth graded as II, III, IV, V, I, tiny VI retrolaterally; I, II, III, IV, tiny V prolaterally (Figs 10G, H, 12F, 15G, H).

***Opisthosoma*.** The entire surface covered with almost adpressed setae and numerous long, curved, bifurcate setae. Genital operculum triangular in shape with no clear demarcation between the plates., and the rear edge of the genital sternite not chitinized (Fig. 17F). Sternite IV with 14 short needle-like ctenidia extending 1/3 the length of the succeeding sternite (Fig. 18E).

***Pedipalps*.** Totally covered with short setae and long, thick setae.

***Legs*.** As in the males.

##### Variability.

Female. Total length 20.13–23.67. Body coloration pale yellow to yellow. The number of cheliceral fixed finger fondal teeth 10–12 (profondal teeth 4–6; retrofondal teeth 6–7). MST 3–5, MSP 3–4. The number of ctenidia on sternite IV 14–16.

##### Distribution and habitat.

China (Xizang). Habitat: grassland (Fig. 2H).

##### Remark.

Based on the comparison of genetic distances, with a genetic distance of 0% (Table 2) between male and female collected from same locations, we believe that they are same species.

## ﻿Discussion

The cytochrome c oxidase subunit I (COI) gene has been extensively employed in taxonomic and differentiation studies of Arachnida species. Analysis of COI sequences enables researchers to deepen their understanding of genetic variances among various species, thus facilitating more precise delineation and differentiation (Maddahi et. al. 2016).

The rich diversity of *Karschia* in Xizang can be ascribed to its distinctive geographical environment and climate conditions. With an average elevation surpassing 4000 meters, Xizang features vast high-altitude lakes and intricate geographical terrain, which hinder species dispersal, resulting in geographic isolation. Furthermore, we documented the highest Solifugae record (*K.shigatse* sp. nov.) in the Old World, complete with precise geographic coordinates, at an elevation of 4605.8 meters.

After examining various taxonomic characteristics of solifuge species and comparative specimens in this research, we think cheliceral teeth possess some taxonomic value, particularly regarding the relative size between the primary teeth of the median teeth series, which remains constant: the fondal series typically exhibit variability, and the numbers of secondary teeth generally have poor taxonomic value unless a specific type of tooth is entirely missing. Body size and coloration are subject to variation, with the opisthosoma being relatively soft and its size susceptible to change based on the nutritional state of the specimen; coloration can fluctuate with environmental changes, indicating that these traits have limited taxonomic value. The numbers of ctenidia on the sternite and spines of the male pedipalp also display variation, albeit to a lesser extent, making them suitable as additional diagnostic characters; however, the shape and size of ctenidia on the sternite are relatively constant, rendering them reliable taxonomic characteristics. The study confirms the significance of the female genital operculum in classification, as the shape, size, and relative arrangement of the genital operculum remain consistent among female individuals of the same species; the flagellar complex of the male serves as reliable diagnostic characteristics, particularly regarding the degree of modification of the *fcp* (flagellar complex process plumose setae), the shape and number of *fcs* (flagellar complex subspiniform to spiniform setae), and the lateral apophysis.

Reevaluating the taxonomy of numerous Karschiidae species is indeed crucial and urgent. Historically, their classification and diagnostic criteria have heavily leaned on the cheliceral teeth traits of only a limited number of female specimens (Birula 1922, 1938; Roewer 1933, 1934). However, this approach may not fully capture the diversity and variation present within these species. Therefore, a more comprehensive assessment that considers a broader range of taxonomic characters, including those mentioned earlier such as the shape and numbers of ctenidia on the sternite and the shape and size of the genital operculum in females, is necessary. By incorporating these additional diagnostic characteristics, we can better elucidate the taxonomy and improve our understanding of the intricate relationships among *Karschia* species.

## Supplementary Material

XML Treatment for Karschia (Karschia) tibetana

XML Treatment for Karschia (Karschia) dingye

XML Treatment for Karschia (Karschia) lhasa

XML Treatment for Karschia (Karschia) zhui

XML Treatment for Karschia (Karschia) shigatse

XML Treatment for Karschia (Karschia) namling

## ﻿Additional information

### Author ORCIDs

Wenlong Fan https://orcid.org/0009-0004-6854-6330

Chao Zhang https://orcid.org/0000-0003-1702-1206

Feng Zhang https://orcid.org/0000-0002-3347-1031

### Data availability

All of the data that support the findings of this study are available in the main text.
